# Deterministic Channel Modeling in Urban Multi-Factor Environments Based on a Hybrid Forward-Backward Ray Tube Tracing Approach

**DOI:** 10.3390/s26175448

**Published:** 2026-08-28

**Authors:** Qi Yao, Zhongyu Liu, Lixin Guo

**Affiliations:** School of Physics, Xidian University, Xi’an 710071, China

**Keywords:** ray tracing, channel modeling, vegetation scattering, bidirectional scattering distribution function, urban environment, deterministic propagation

## Abstract

Deterministic channel models are essential for high-frequency communication system design in complex urban environments, where multiple propagation mechanisms including reflection, diffraction, and vegetation scattering coexist. This paper proposes a hybrid forward–backward ray tube tracing (HFB-RTT-3D) approach that extends the established ray tracing fusion with multiple diffuse scattering (RT-MDS) framework from natural terrain to urban scenarios by introducing pyramid-shaped diffraction and vegetation scattering ray tubes. A vegetation scattering model based on a leaf-level bidirectional scattering distribution function (BSDF) is established, enabling computationally feasible representation of vegetation effects in deterministic channel prediction. The framework thereby covers buildings, vegetation, and terrain within a unified ray tube data structure. Simulation analyses quantify vegetation modulation of multipath structure and received power across frequencies from 5 to 15 GHz and different canopy sizes. Measurement validation in a campus tree-lined avenue scenario at 2.3 to 5.9 GHz demonstrates that incorporating vegetation scattering reduces the root mean square error (RMSE) of the prediction to 6.11 to 6.30 dB, an improvement of 0.57 to 1.67 dB over the case without vegetation, confirming the effectiveness of the proposed method.

## 1. Introduction

### 1.1. Research Background and Significance

As fifth-generation and sixth-generation mobile communication systems evolve toward millimeter-wave and even terahertz frequency bands, wireless channel modeling faces unprecedented challenges in terms of accuracy and dimensionality [1,2]. High-frequency electromagnetic wave propagation exhibits prominent characteristics including narrow beamwidth, weak diffraction capability, and high penetration loss, rendering traditional statistical channel models incapable of accurately capturing multipath structures and space-time-frequency characteristics in specific scenarios [3]. Deterministic channel models have therefore become a key tool for supporting high-frequency system design and performance evaluation [4].

Among representative 5G and 6G application scenarios, millimeter-wave mobile broadband (MMB) systems, device-to-device (D2D) communications, and vehicle-to-everything (V2X) communications are particularly relevant to the present work. In an MMB system, several access points are deployed in a high-density indoor or hotspot area and are interconnected through wireless millimeter-wave backhaul links, in order to satisfy the multi-gigabit data rate demand and to relieve the load on the wired core network [5]. D2D communications, on the other hand, enable direct transmissions between nearby user equipments without traversing the base station, which can substantially reduce the end-to-end latency and the energy consumption of mobile devices while offloading the cellular infrastructure [5]. V2X communications extend the application landscape toward cooperative automated driving and road-safety services, in which vehicles, roadside infrastructure, and pedestrians exchange safety-critical messages along urban streets and tree-lined avenues [6]. In such scenarios, the links frequently propagate through roadside vegetation and along building facades, so that the multipath structure, the blockage probability, and the shadowing statistics are governed by the same site-specific propagation mechanisms that the proposed framework is designed to reproduce. These scenarios place deterministic channel modeling at a central position, because the link-level performance of MMB backhaul links, the interference pattern among coexisting D2D pairs, and the outage probability in shadowed urban regions are all sensitive to the multipath structure, the delay and angular spreads, and the vegetation-induced attenuation that can only be faithfully reproduced by a site-specific ray-based engine. The unified treatment of specular reflection, diffuse scattering, edge diffraction, and vegetation scattering within a single ray-tracing framework is therefore a prerequisite for the reliable design and performance evaluation of these systems in complex urban environments.

Urban hotspot areas, as the most traffic-intensive scenarios in mobile communications, present highly complex propagation environments [7]. Dense building clusters generate abundant reflection, diffraction, and penetration paths; areas with roadside trees and green spaces introduce additional scattering and attenuation effects; and the penetration loss mechanisms in indoor–outdoor transition zones interact [8,9]. These coexisting multi-factor complex propagation environments impose higher demands on deterministic channel modeling approaches, requiring not only efficient three-dimensional ray tracing algorithms to capture major propagation paths but also refined environmental electromagnetic models to characterize various scattering and attenuation mechanisms.

### 1.2. Current Research Status and Analysis

Traditional forward ray tracing methods face two key challenges: computational redundancy and path omission [4,10]. Launching numerous rays at fixed angular resolution from the transmitter (Tx) leads to most rays delivering negligible power at the receiver (Rx) after several reflections, causing significant computational inefficiency. Simultaneously, limited by ray tube resolution and intersection testing accuracy, some effective paths arriving at the Rx may be missed due to discrete sampling of ray tubes [11], especially when handling propagation mechanisms such as diffraction and scattering that require high angular resolution support.

Existing urban environmental channel models address vegetation scattering effects in a simplified manner. Most models treat vegetation regions as homogeneous or random media with certain attenuation coefficients [12], employing empirical attenuation models to approximate vegetation penetration loss, while lacking a complete physical characterization of the directional and frequency-dependent nature of vegetation scattering [8,13]. Although leaf-level bidirectional scattering distribution function (BSDF)-based methods have been well-developed in the remote sensing field [14], their application in wireless channel modeling remains at the exploratory stage. How to integrate the BSDF scattering model into a three-dimensional ray tracing framework in a computationally feasible manner remains a key problem to be solved.

Image-based methods and ray launching methods each have limitations in deterministic propagation modeling [15,16]. Image-based methods offer advantages of high precision and efficiency when handling planar reflections, generating accurate specular reflection paths. However, they lack natural extensibility for non-specular propagation mechanisms such as curved surfaces, edge diffraction, and diffuse scattering. Ray launching methods are more general and can naturally handle multiple interactions of complex geometries, but their path extraction accuracy is limited by ray density, requiring a substantial number of rays to ensure convergence in high-precision channel prediction applications [10,17].

### 1.3. Research Foundation and Main Contributions

The work in this paper represents a systematic extension and refinement of the ray tracing fusion with multiple diffuse scattering (RT-MDS) framework published in our previous work [18]. Prior work has validated the effectiveness of this architecture in fusing high-order diffuse scattering in three-dimensional mountainous terrain environments and improving ray-scene collision computation efficiency by a factor of approximately 16 through the voxelized signed distance field (VSDF) acceleration method. However, the model still lacks comprehensive physical characterization of propagation mechanisms such as vegetation scattering and edge diffraction that are prevalent in urban environments. To this end, while inheriting the same ray tube tree data structure and forward-backward tracing framework, this paper systematically extends the modeling capability from natural terrain to urban hotspot areas by introducing construction modules for pyramid-shaped diffraction ray tubes and vegetation scattering ray tubes, and establishing refined environmental geometric and electromagnetic models encompassing buildings and vegetation.

The main contributions of this paper include the following three aspects. First, a unified construction method for edge diffraction and vegetation BSDF scattering ray tubes tailored to urban environments is proposed, achieving collaborative modeling of four propagation mechanisms, namely, specular reflection, diffuse scattering, edge diffraction, and vegetation scattering, within a single ray tube tree framework. Second, a vegetation scattering model based on the leaf-level BSDF is established, with the BSDF serving as the core physical model to achieve computationally feasible representation of vegetation effects in deterministic channel prediction without explicitly modeling vegetation micro-geometry. Third, deterministic channel prediction is extended from mountainous terrain scenarios to urban multi-factor environments encompassing buildings and vegetation, and the effectiveness of the proposed method is validated through measured data at four frequency points from 2.3 to 5.9 GHz in a campus tree-lined avenue scenario.

## 2. Urban Environment Characterization and Modeling

This section builds upon the three-dimensional undulating terrain environment modeling method established in the RT-MDS framework [18] by introducing two major urban environmental elements, buildings and vegetation, extending the applicable scenarios of deterministic channel modeling from natural terrain to urban hotspot areas.

### 2.1. Building Structure Modeling

Building modeling is differentiated into two strategies, namely, refined and non-refined, according to scenario requirements.

For large-scale urban-level simulation scenarios where precise characterization of building surface details is not required, a lightweight modeling approach based on OpenStreetMap contour extrusion is adopted [19,20].

**Figure 1:**
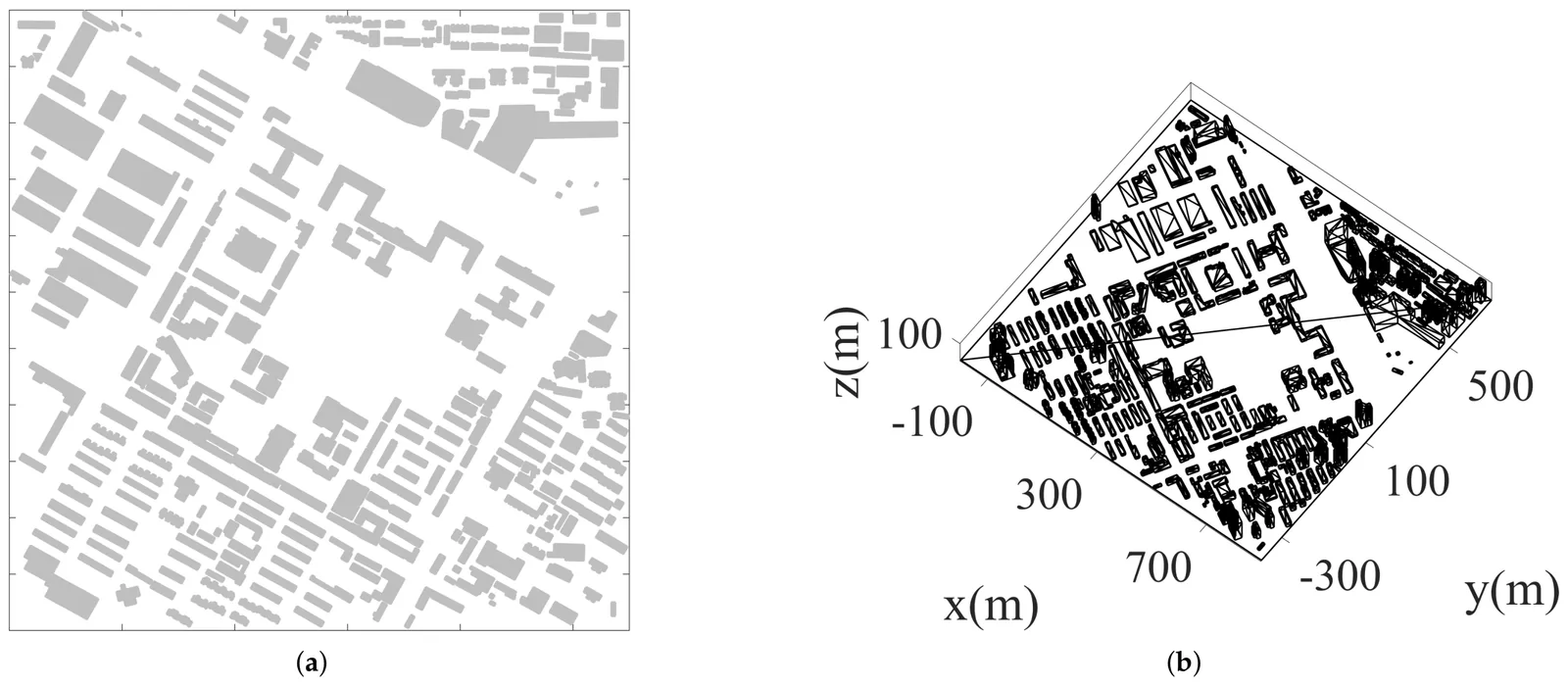
Building modeling based on OpenStreetMap. (**a**) Raw building footprint polygons exported from OpenStreetMap for the campus region, rendered in greyscale with all buildings shown in a single colour; the per-building height is recovered from the stored height attribute in the subsequent extrusion step. (**b**) The resulting triangulated three-dimensional scene after the three processing steps of contour regularization, vertical extrusion by the height attribute, and triangulation of the polyhedral surfaces, rendered as a wireframe viewed from an oblique overhead angle, with the *x*, *y*, and *z* coordinate axes overlaid to indicate the spatial orientation.

As shown in Figure 1a, OpenStreetMap vector data provide building footprint polygons, each defined by a set of vertex sequences with geographic coordinates and associated with attribute information such as building height and number of floors. The height of each polygon is read from the stored building height attribute, and it is this height value that determines the elevation of the corresponding polyhedron in Figure 1b after extrusion. The modelling process first simplifies and regularises the original contours to eliminate minor serrations and redundant vertices, then extrudes the two-dimensional polygons into three-dimensional polyhedra along the vertical direction according to height attributes, and finally discretises the polyhedral surfaces into triangular meshes through triangulation, with the result shown in Figure 1b. This strategy preserves large-scale geometric features such as overall building volume, orientation, and spatial occupancy while omitting surface details such as window sills, balconies, and curtain wall divisions, controlling the facet count of individual buildings on the order of hundreds to thousands. In square-kilometre-scale scenarios containing tens of thousands of buildings, the total number of environmental facets is controlled within a million-level range, ensuring that forward ray tube tree generation and backward path retrieval are completed within an acceptable engineering timeframe, satisfying the simulation efficiency requirements for city-level coverage planning.

**Figure 2:**
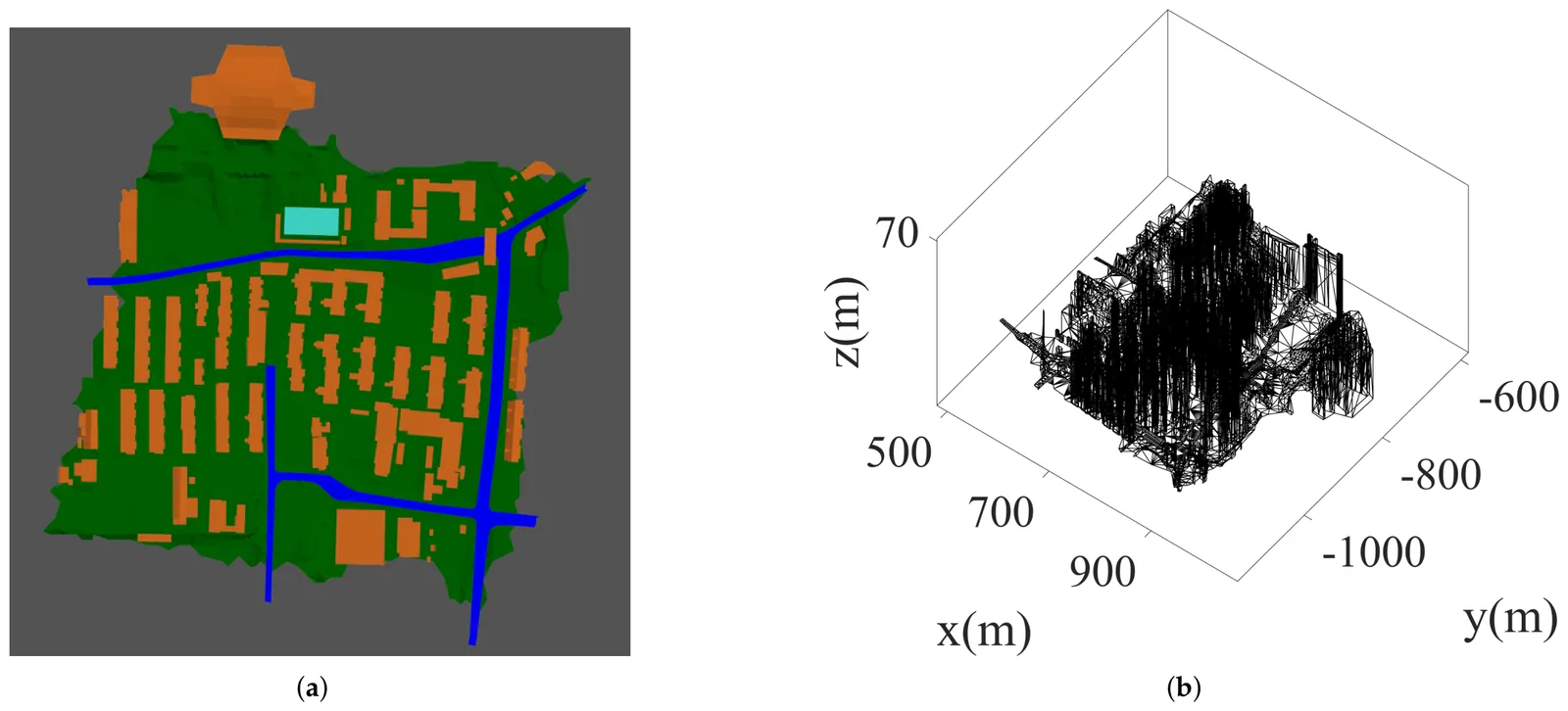
Building modeling based on point cloud data. (**a**) Classified airborne LiDAR point cloud of the same campus region shown in Figure 1, with vegetation, buildings, and roads rendered in distinct colours. (**b**) Triangulated mesh model reconstructed from the point cloud through normal vector estimation and screened Poisson surface reconstruction, rendered as a dense wireframe with the *x*, *y*, and *z* coordinate axes overlaid to indicate the spatial orientation.

For key areas requiring higher geometric accuracy, a refined triangular mesh reconstruction method based on airborne Light Detection and Ranging (LiDAR) point cloud data is adopted [21]. After filtering and classification, the point cloud yields building point sets, as shown in Figure 2a, which are converted into triangular mesh models, as shown in Figure 2b, through normal vector estimation and Poisson surface reconstruction [21]. The facet count of refined models is typically tens to hundreds of times that of contour extrusion models. In practical applications, this strategy is employed only for key building clusters within the first Fresnel zone around the Tx, enhancing prediction accuracy in critical areas while maintaining overall simulation feasibility. The two methods can be flexibly combined to form an adaptive modelling framework spanning broad coverage and localised high-fidelity refinement. It must be emphasised that, for both Figure 1b and Figure 2b, the fundamental geometric unit is the triangle, and every triangle supports the independent assignment of material electromagnetic parameters.

### 2.2. Vegetation Parametric Modeling

**Figure 3:**
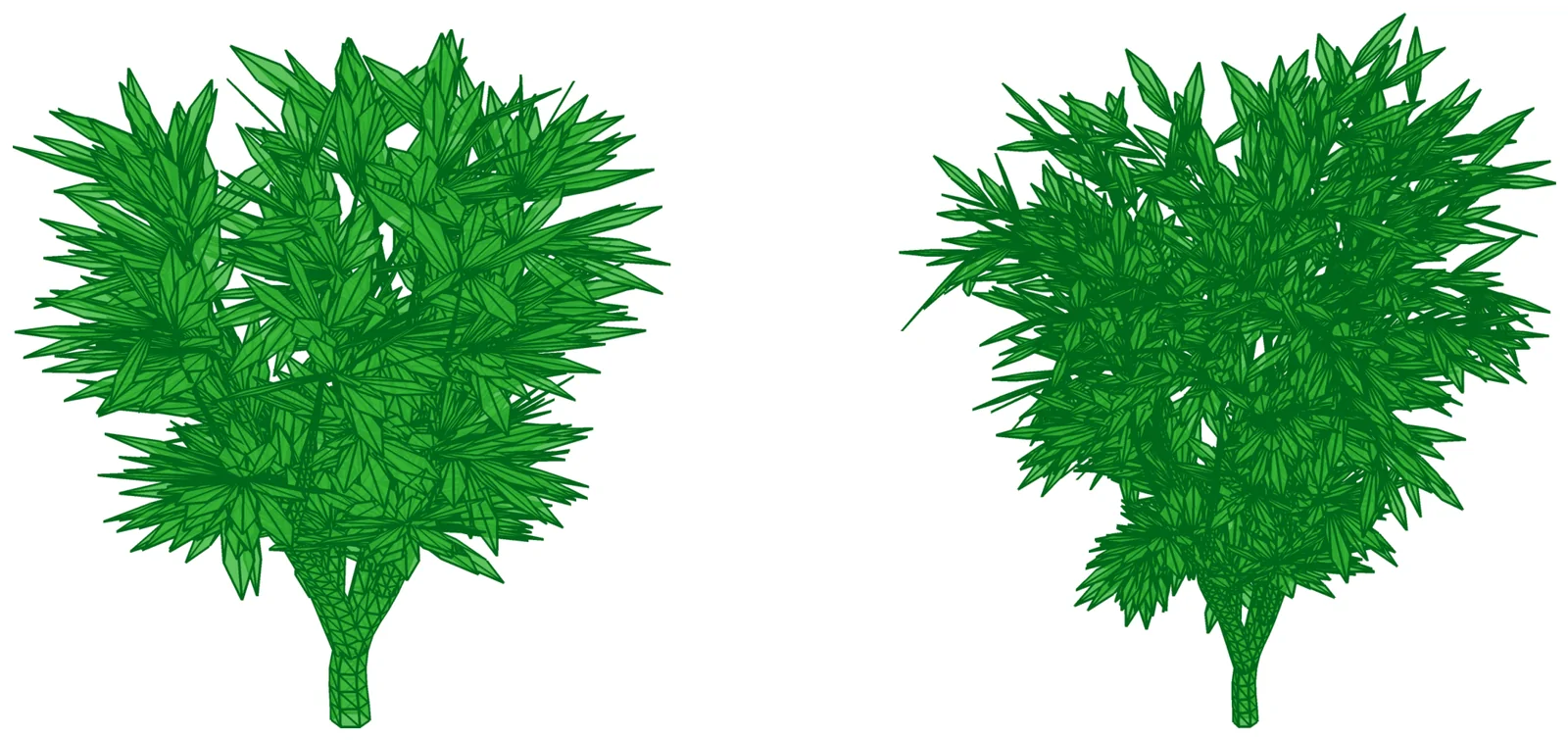
Geometric models of two different tree types.

In wireless channel prediction for urban hotspot areas, vegetation, as a natural element with irregular morphology and complex electromagnetic characteristics, exerts non-negligible influence on wave propagation and must therefore be represented within the ray tracing framework using a three-dimensional vegetation model that balances geometric realism with computational feasibility. This paper employs a procedural generation method driven by L-system (Lindenmayer system) rules [22], which efficiently generates statistically plausible geometric tree proxies from limited parameter inputs such as species, tree height, crown width, and branching levels, offering both flexibility and reproducibility. Notably, high-fidelity triangular mesh models obtained through LiDAR point cloud data fusion and surface reconstruction can also serve as vegetation inputs for this work. To systematically characterize the three-dimensional tree geometry generated procedurally, the geometric parameter vector defined in this paper is as follows:

(1) $$\begin{array}{c} P_{geo}=\{Shape,Levels,\theta_{branch},Length,Curvature,Taper,Prune,\\ BranchDist,LAI,H,R_{base},L_{leaf},W_{leaf},Seed\} \end{array}$$

where Shape is the tree shape template (conical, spherical, or elliptical), Levels controls branch hierarchy depth, $\theta_{branch}$ is the branching angle, Length and Curvature control branch length and bending degree, respectively, Taper is the radius attenuation rate, Prune is the Boolean pruning flag, BranchDist is the vertical branch distribution density, LAI is the leaf area density, *H* and $R_{base}$ control tree height and base radius ratio, $L_{leaf}$ and $W_{leaf}$ are leaf dimensions, and Seed is the random generation seed. Geometric models of two trees with different parameters are shown in Figure 3.

In this paper, to maintain consistency with environmental elements such as terrain and buildings, relative permittivity and conductivity are selected to characterize the electromagnetic parameters of vegetation. For example, at 2.2 GHz [14], the relative permittivity of moist lemon leaves is 23.26 and the conductivity is 0.89 S/m. At 75 GHz [12], the relative permittivity of dry leaves is approximately 1.5 with extremely low conductivity, close to that of an ideal insulator, and is set to $1\times 10^{-8}$ S/m in the simulations of this paper. The vegetation electromagnetic model parameter vector defined in this paper is:

(2) $$P_{em}=\left\{P_{geo},\varepsilon_{r}\left(f\right),\delta \left(f\right)\right\}$$

where *f* is the frequency, $\varepsilon_{r}\left(f\right)$ is the relative permittivity of vegetation at the corresponding frequency, and δ(f) is the conductivity of vegetation at the corresponding frequency. Because the frequency dependence of vegetation permittivity and conductivity originates from the dielectric relaxation of bound water and free water within the leaf and branch tissues, both $\varepsilon_{r}\left(f\right)$ and δ(f) exhibit a monotonically decreasing trend in the microwave band and a near-constant behaviour at millimetre-wave and sub-terahertz frequencies, with the transition being mainly controlled by the vegetation water content and the relaxation time constant. In the present work, $\varepsilon_{r}\left(f\right)$ and δ(f) are not expressed by a closed-form analytical dispersion formula; instead, they are assigned by discrete values taken from peer-reviewed measurement data [12,14] at the frequencies of interest, and a linear interpolation in the logarithmic domain is applied between adjacent sample frequencies. This look-up-table-based representation is sufficient for the simulation frequencies considered in this paper (5 to 15 GHz for the synthetic scenario and 2.3 to 5.9 GHz for the measurement scenario) and avoids the introduction of additional fitting parameters. The discrete permittivity and conductivity values directly enter the leaf-level scattering coefficient model described in Section 3.3.1, and through the BSDF they govern the magnitude and angular distribution of the vegetation scattered energy that contributes to the deterministic multipath prediction.

In the wireless channel simulation scenarios constructed in this paper, all environmental elements including terrain, buildings, and vegetation share a common fundamental unit: triangular facets with electromagnetic material attributes [23]. This strategy offers three advantages: it supports independently assigned electromagnetic parameters for each facet, reflecting the spatial heterogeneity of electromagnetic characteristics in real environments; triangular meshes, as a universal geometric representation, can accommodate multi-source input data including manual modeling, procedural generation, and LiDAR reconstruction; and geometric and electromagnetic information are tightly coupled within the same primitive, allowing direct retrieval of material parameters during ray intersection, enabling energy-domain pruning optimization and significantly improving simulation efficiency while ensuring accuracy.

## 3. Propagation Model Construction Method

The pyramid-shaped ray tube data structure, the recursive generation logic of the ray tube tree, and the core forward-tree building and backward-path-retrieval tracing strategy adopted in this paper remain consistent with the RT-MDS model established by the authors [18]. While briefly restating this general framework, this section focuses on elaborating the construction methods for diffraction and vegetation scattering ray tubes newly introduced to accommodate urban environments. Pyramid-shaped transmitting ray tubes, pyramid-shaped reflection ray tubes, and pyramid-shaped diffuse scattering ray tubes have been described in detail in RT-MDS and are not repeated here. The proposed hybrid forward-backward ray tube tracing approach (HFB-RTT-3D), targeting buildings, vegetation, and other urban environmental elements, introduces construction methods for pyramid-shaped diffraction ray tubes and pyramid-shaped vegetation scattering ray tubes based on RT-MDS. The addition of these two new ray tube types enables the framework to comprehensively characterize propagation mechanisms critical in urban scenarios.

Throughout the synthetic simulations reported in this paper, both the transmitter and the receivers are modeled as ideal isotropic radiators with unit gain, linear vertical polarization, and a conjugate-matched receive chain. This choice is deliberate: the goal of the present work is to characterise the propagation channel itself, not any specific antenna front-end, so that the simulation results can be reused as an input by any subsequent system-level simulation. In the measurement campaign, a biconical omnidirectional antenna (model SZ-2001800/P) is used at both the transmitter and the receiver, whose omnidirectional radiation in the horizontal plane is consistent with the isotropic assumption adopted in the simulation; the antenna type and its gain are documented together with the frequency plan in Section 4, and the same frequency-dependent path loss and angular spread are observed in both simulation and measurement, which confirms that the antenna assumption does not contaminate the comparison. The transmit power, the carrier frequency, the receiver height, and the receiver grid spacing used in each simulation case are listed in the corresponding subsection of Section 4.

### 3.1. Complete Model Construction Workflow

**Figure 4:**
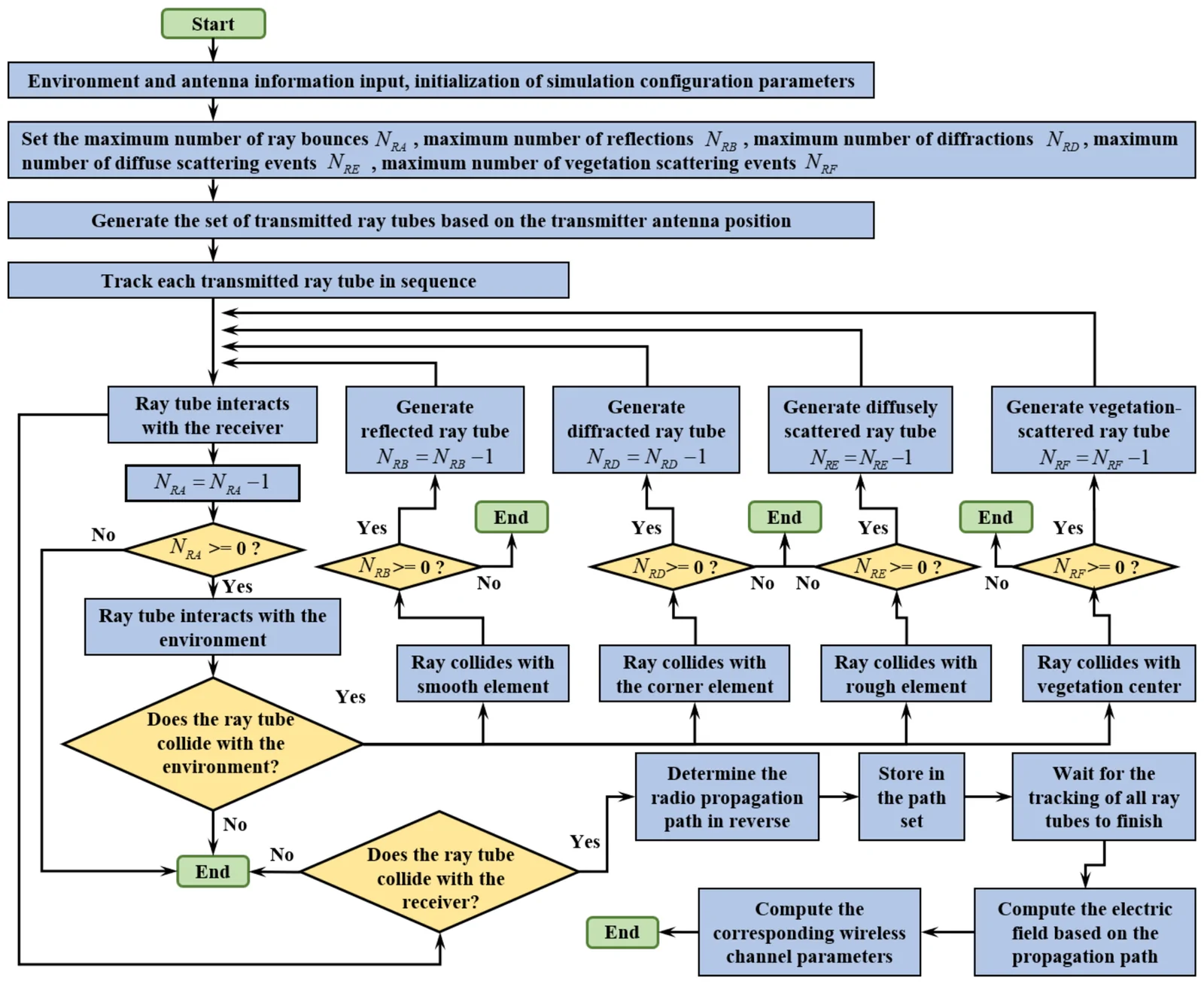
Construction workflow of HFB-RTT-3D.

The HFB-RTT-3D workflow, as illustrated in Figure 4, constitutes a systematic, iterative computational process. Initially, the geometric and electromagnetic information of the three-dimensional urban environment and simulation control parameters (maximum number of bounces, reflections, diffractions, diffuse scattering, and vegetation scattering events) are input, and the initial transmitting ray tubes are added to a pending queue. In the main loop, ray tubes are retrieved from the queue one by one and sequentially undergo path detection (whether they directly reach the Rx), collision detection (effective collisions with facets and edges), and bounce counter updating.

Based on the geometric properties and material types of collision results, new ray tubes are generated: a reflection ray tube is generated upon collision with a smooth facet, a diffraction ray tube is generated according to the method in Section 3.2 upon collision with an edge, a diffuse scattering ray tube is generated upon collision with a rough facet, and a vegetation scattering ray tube is generated according to the method in Section 3.3 upon collision with a vegetation facet. All new ray tubes inherit the remaining counters of the current ray tube and are added to the queue. After the pending queue is emptied, backward tracing is performed for all successful paths to determine complete propagation sequences, and the complex gain of each path is computed to output channel parameters. This extended workflow achieves comprehensive coverage modeling of complex propagation mechanisms in urban hotspot areas through the introduction of two new ray tube types, diffraction and vegetation scattering, while controlling computational complexity through coordinated multi-counter constraints.

### 3.2. Construction of Pyramid-Shaped Diffraction Ray Tubes

**Figure 5:**
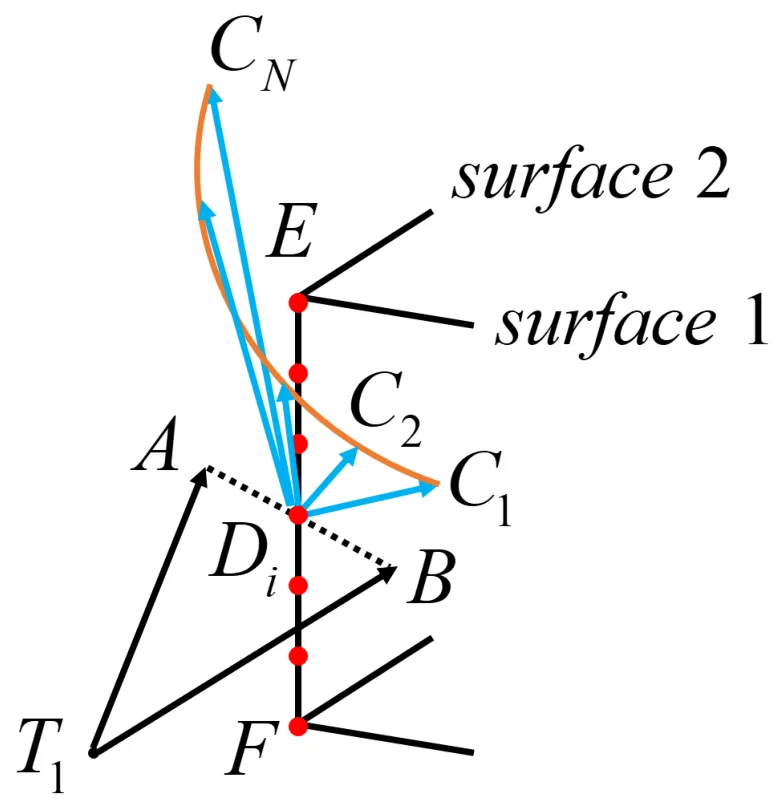
Schematic diagram of pyramid-shaped diffraction ray tube construction.

In urban hotspot areas, building edges, roof eaves, and edges of street furniture are key diffraction sources generating non-line-of-sight (NLoS) propagation. When a pyramid-shaped ray tube being traced makes a valid collision with a predefined edge in the scene and satisfies visibility determination conditions, the algorithm triggers the diffraction ray tube construction process. As shown in Figure 5, when ray tube $T_{1}-h-\vec{T_{1}A}-\vec{T_{1}B}$ collides with edge EF and the diffraction center $D_{i}$ is illuminated by $T_{1}-h-\vec{T_{1}A}-\vec{T_{1}B}$, diffraction ray tubes $D_{i}-h-\vec{D_{i}C_{1}}-\vec{D_{i}C_{2}}$, $D_{i}-h-\vec{D_{i}C_{2}}-\vec{D_{i}C_{3}}$, …, $D_{i}-h-\vec{D_{i}C_{N-1}}-\vec{D_{i}C_{N}}$ are generated.

The diffraction center $D_{i}$ in Figure 5 is computed according to the preset maximum diffraction segment length $l_{d}$ in this paper, with the following formula:

(3) $$\begin{array}{c} D_{i}=E+\left(i-0.5\right)l_{d_{1}}\hat{v}\;,\;i=1,2,\ldots ,m\\ m=[|\vec{EF}|/l_{d}]+1\\ l_{d_{1}}=\left|\vec{EF}\right|/m \end{array}$$

where *E* and *F* are the two vertices of the common edge of surface1 and surface2, $\hat{v}$ is the unit vector of $\vec{EF}$, and the rays $\vec{D_{i}C_{k}}$ (k=1,2,…,N) emitted by the generated diffraction ray tube are determined by the diffraction range and discrete angle magnitude. The discretization process is similar to that of pyramid-shaped diffuse scattering ray tubes. The newly generated diffraction ray tube inherits all propagation attributes of the current ray tube except the diffraction count, with its remaining diffraction counter decremented by 1, and is added to the pending queue. This mechanism ensures that the model can systematically capture multiple diffraction and reflection–diffraction hybrid paths caused by building edges, significantly improving coverage prediction accuracy in shadow regions such as urban canyons.

### 3.3. Construction of Pyramid-Shaped Vegetation Scattering Ray Tubes

In urban hotspot areas, vegetation (such as roadside trees, shrubs, and green belts), although geometrically much smaller than buildings, produces intense scattering and attenuation effects on high-frequency electromagnetic waves through its dense branch-and-leaf structure. If every leaf and branch were to be explicitly modeled geometrically, the facet count would reach millions or even hundreds of millions, rendering ray tracing computation infeasible within an engineering timeframe. Therefore, this model abandons direct tracing of vegetation micro-geometry and instead adopts an equivalent scatterer approach: each individual tree or vegetation cluster is abstracted as a central point with statistical scattering characteristics, and the BSDF is employed to describe the spatial redistribution of energy after incident rays interact with vegetation.

The replacement of an explicit branch-and-leaf geometry with a single equivalent scattering centre per tree is a deliberate approximation, and its physical justification rests on three observations. First, at the frequencies considered in this work (2.3 to 15 GHz), the wavelength is much larger than the typical leaf dimension and is comparable to or larger than the inter-branch spacing, so the canopy behaves as a volumetric scattering medium rather than a collection of isolated scatterers. Second, the first-order coherent contribution of the canopy is concentrated in the forward direction and can be represented as a complex transmission coefficient through the canopy, while the incoherent component is distributed over a broad solid angle and is well captured by a rotationally symmetric BSDF. Third, the main observable of the deterministic channel model is the set of power angular spectra and the received power, both of which are integral quantities over the canopy volume and are insensitive to the precise internal arrangement of the scatterers. Under these conditions, a single equivalent centre with a pre-computed BSDF is the minimal representation that preserves the angular and frequency dependence of the canopy response. A sensitivity analysis that varies the number of equivalent centres per tree and their spatial arrangement is identified as future work and will be reported in a separate study, because it requires dedicated measurement campaigns on vegetation clusters with different ages, species, and pruning conditions that are not within the scope of the present paper.

#### 3.3.1. Vegetation BSDF

The bidirectional scattering distribution function (BSDF) is the central physical quantity that couples the leaf-level electromagnetic model to the ray-tracing engine. Following the radiometric definition used in computer graphics and in remote sensing, the BSDF is defined as the ratio between the scattered spectral radiance and the incident spectral irradiance:

(4) $$f\left(\theta_{i},\varphi_{i};\theta_{s},\varphi_{s}\right)=\frac{L_{s}\left(\theta_{s},\varphi_{s}\right)}{E_{i}\left(\theta_{i},\varphi_{i}\right)}$$

where $\left(\theta_{i},\varphi_{i}\right)$ and $\left(\theta_{s},\varphi_{s}\right)$ denote the incident and scattering directions, respectively, $E_{i}$ is the incident spectral irradiance on the vegetation element, and $L_{s}$ is the scattered spectral radiance leaving the vegetation element in the direction $\left(\theta_{s},\varphi_{s}\right)$. The BSDF has the unit of inverse steradian and is independent of the absolute incident power level, which makes the scattered energy of a vegetation element scale linearly with the incident ray tube power.

For a passive vegetation scatterer, the discretised BSDF must satisfy the hemispherical energy conservation constraint

(5) $$\sum_{k}f\left(\theta_{i},\varphi_{i};\theta_{s,k},\varphi_{s,k}\right)\cos\left(\theta_{s,k}\right)\Delta \Omega_{s,k}\leq 1$$

with equality only in the lossless limit. The inequality expresses the physical requirement that the canopy cannot amplify the incident radiation, and it is strictly enforced during the pre-simulation of the vegetation material library. As a result, the BSDF entry that is finally stored on disk can only attenuate or, in the lossless limit, conserve the incident energy.

The numerical procedure used to obtain $f(\theta_{i},\varphi_{i};\theta_{s},\varphi_{s})$ consists of three steps. (i) For a representative vegetation element with a given leaf-area density, leaf dimensions, and branch architecture, the leaf-level scattered field is computed under a plane-wave excitation at the carrier frequency. The excitation uses the relative permittivity $\varepsilon_{r}\left(f\right)$ and conductivity δ(f) assigned by the discrete look-up table described in Section 2.2. (ii) The scattered field is integrated over a far-field sphere centered on the vegetation element, yielding a dense angular grid of scattered power. (iii) The BSDF on this dense grid is convolved with the angular footprint of the incident ray tube and re-sampled on the coarser $\left(\theta_{i},\varphi_{i};\theta_{s},\varphi_{s}\right)$ grid used at runtime. In the present paper, the angular discretisation interval of the incident direction is 6° and that of the scattering direction is 1°.

The discretized BSDF is stored as a vegetation material library entry, indexed by frequency, polarization, and vegetation species. At runtime, when a ray tube makes a valid collision with a vegetation centre point, the algorithm retrieves the BSDF entry that matches the current frequency and polarization, uses it to weight the energy of the newly generated scattering ray tubes, and emits them in a set of discrete directions $\left(\theta_{s,k},\varphi_{s,k}\right)$ over the full 4π solid angle, with the weight of each direction given by $f\left(\theta_{i},\varphi_{i};\theta_{s,k},\varphi_{s,k}\right)\cos\left(\theta_{s,k}\right)$. Because the BSDF is pre-computed once per material entry and is only retrieved at runtime, the per-collision overhead of the vegetation scattering mechanism is reduced to a memory access and a small number of multiply-accumulate operations, which is essential for keeping the wall-clock time of the deterministic engine compatible with the engineering constraint of processing tens of thousands of receivers per scenario.

#### 3.3.2. Generation of Pyramid-Shaped Vegetation Scattering Ray Tubes

**Figure 6:**
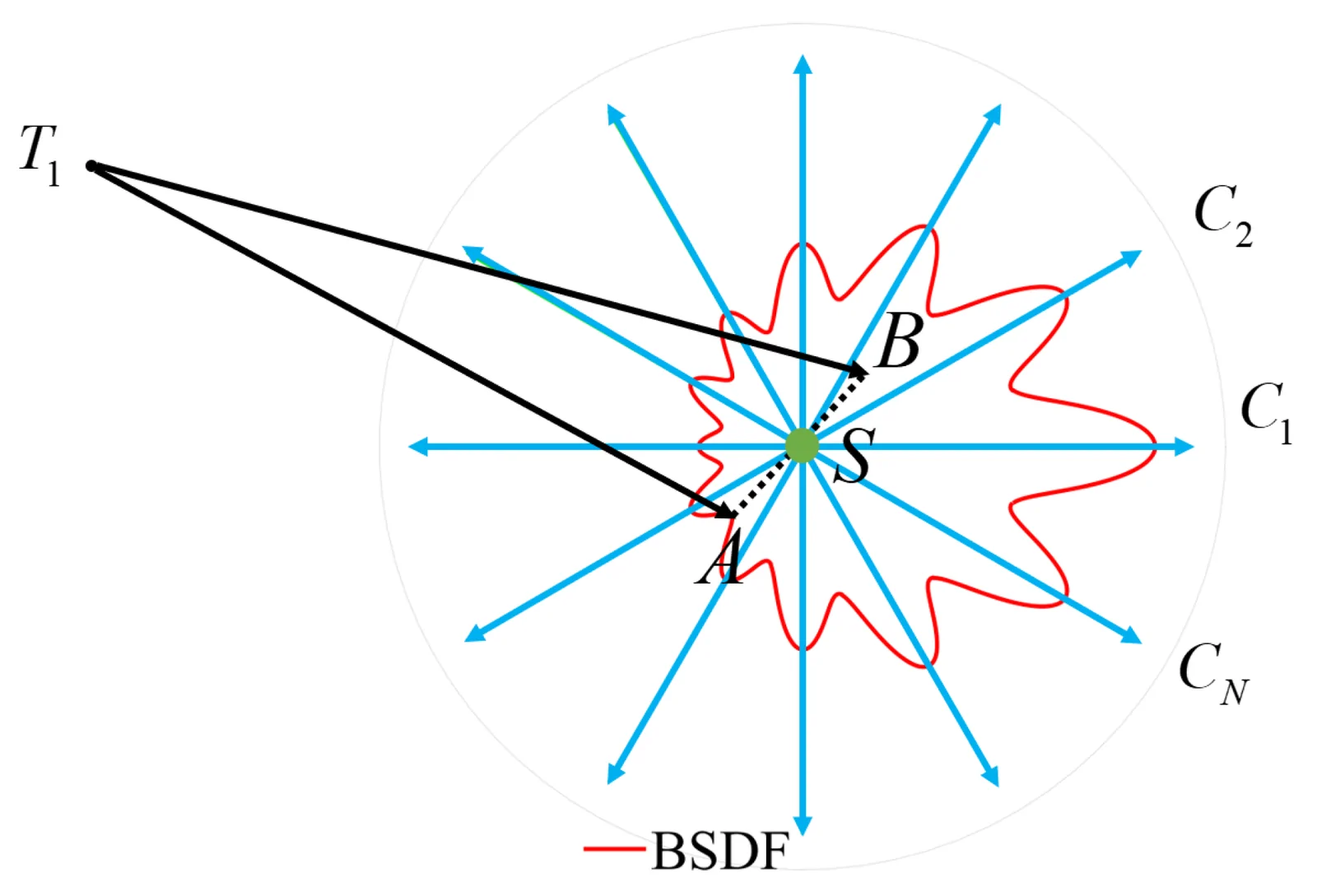
Schematic diagram of pyramid-shaped vegetation scattering ray tube construction.

When a pyramid-shaped ray tube being traced makes a valid collision with a predefined vegetation center point in the scene, the algorithm triggers the pyramid-shaped vegetation scattering ray tube construction process, as illustrated in Figure 6.

The rays $\vec{SC_{k}}$ (k=1,2,…,N) emitted by the generated vegetation scattering ray tube are determined by the scattering pattern and discrete angle magnitude. The discretization process is similar to that of pyramid-shaped diffuse scattering ray tubes. The newly generated vegetation scattering ray tube inherits all propagation attributes of the current ray tube except the vegetation scattering count, with its remaining vegetation scattering counter decremented by 1, and is added to the pending queue. This mechanism ensures that the model can systematically capture multiple scattering and reflection-scattering hybrid paths caused by vegetation. Without explicitly modeling vegetation micro-geometry, this model efficiently integrates vegetation scattering effects into HFB-RTT-3D using the BSDF as the core physical model, significantly enhancing both the physical completeness and computational feasibility of channel prediction for urban hotspot areas.

### 3.4. Accurate Path Reconstruction via Tree-Structured Backward Tracing

**Figure 7:**
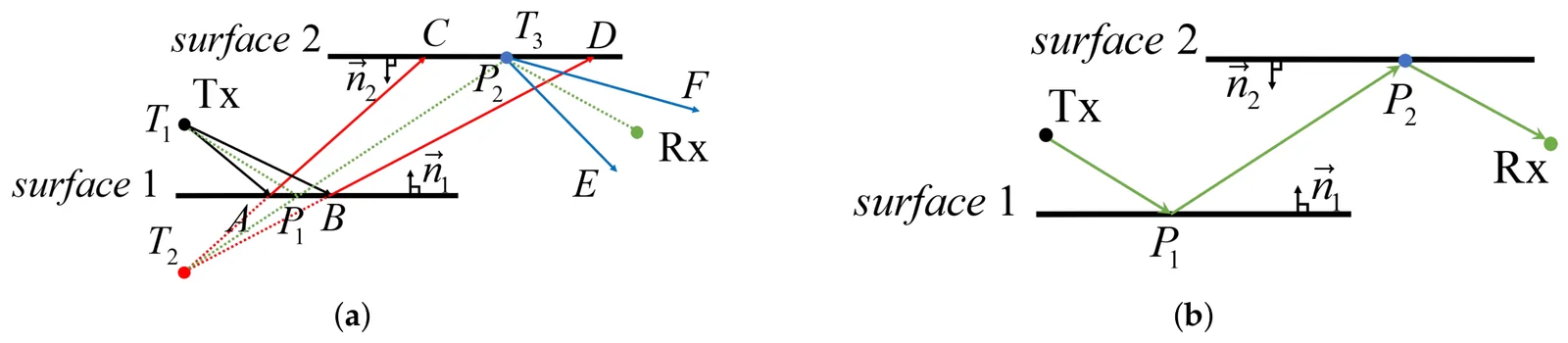
Schematic diagram of backward path determination: (**a**) propagation process; (**b**) path result.

In the forward construction process described above, each ray tube records its parent ray tube information at the time of generation, thereby forming a ray tube tree rooted at the initial transmitting ray tube with child ray tubes produced at each interaction as nodes. After all forward tracing is completed, all ray tubes that successfully reach the Rx constitute the leaf nodes of this tree. The backward path determination process starts from these leaf nodes, ascends level by level along parent pointers to the root node, and sequentially reads the propagation mechanism type (direct, reflection, diffraction, diffuse scattering, or vegetation scattering) and its geometric parameters recorded at each node during the backtracking process, thereby reconstructing the complete propagation sequence. Figure 7a provides an example involving reflection and diffuse scattering: starting from the Rx, the vertex of the diffuse scattering ray tube and the vertex of the reflection ray tube are connected sequentially back to the Tx, and the connection points between path segments are accurately determined according to the geometric constraints of ray tubes and interacting facets. Compared with the reception sphere method used in the traditional shooting and bouncing ray (SBR) technique, this approach can accurately restore the spatial trajectory of paths, avoiding path distortion caused by discrete sampling.

Figure 7a provides an example of a propagation process involving reflection and diffuse scattering, and the corresponding backward determination result is shown in Figure 7b, with the propagation sequence being Tx $\to P_{1}$ (reflection) $\to P_{2}$ (diffuse scattering) →Rx. According to the physical characteristics of ray tubes, the backward path nodes of transmitting, diffraction, and diffuse scattering ray tubes coincide with their vertices, whereas reflection ray tubes require path node computation through line segment intersection based on interface characteristics. This backward determination method based on the ray tube tree can accurately restore the spatial trajectory of each path, avoiding sequence confusion caused by path branching in forward tracing, and constitutes the key component of this hybrid algorithm for achieving high-precision channel prediction.

### 3.5. Mathematical Definition of Coverage Metrics

To make the coverage-oriented analysis in Section 4 quantitative and reproducible, two scalar metrics are formally defined in this subsection. Let $R={\left\{\left(x_{n},y_{n},z_{n}\right)\right\}}_{n=1}^{N}$ denote the prediction plane sampled on a uniform grid of *N* receiver points, with grid spacing Δx=Δy=1.0 m and prediction height $z_{n}=z_{0}$. For each receiver *n*, the deterministic engine produces a complex impulse response

(6) $$h_{n}\left(\tau \right)=\sum_{l=1}^{L_{n}}a_{n,l}e^{j\phi_{n,l}}\delta \left(\tau -\tau_{n,l}\right)$$

from which the narrow-band received power is

(7) $$P_{r,n}=\sum_{l=1}^{L_{n}}|a_{n,l}{|}^{2}$$

The coverage ratio is then defined as the fraction of receivers whose received power exceeds a problem-dependent threshold $P_{\mathrm{th}}$:

(8) $$\eta_{\mathrm{cov}}=\frac{1}{N}\sum_{n=1}^{N}1_{\left\{P_{r,n}\geq P_{\mathrm{th}}\right\}}$$

where $1_{\left\{\cdot \right\}}$ is the indicator function. The mean received power is

(9) $${\bar{P}}_{r}=\frac{1}{N}\sum_{n=1}^{N}P_{r,n}$$

and is reported in dBm as $10\log_{10}\left({\bar{P}}_{r}/1\;\mathrm{mW}\right)$. The same expressions are used in the tables of Section 4. The choice of $P_{\mathrm{th}}=-160$ dBm in the synthetic studies of Section 4 is the same threshold as in our previous RT-MDS study, so that the two sets of results are directly comparable.

The coverage analysis addressed in this paper is a coverage evaluation rather than an optimisation: given a fixed total radiated power and a fixed set of Tx positions, the proposed HFB-RTT-3D engine evaluates the coverage ratio $\eta_{\mathrm{cov}}$ and the mean received power ${\bar{P}}_{r}$ over the prediction plane. An increase in $\eta_{\mathrm{cov}}$ and ${\bar{P}}_{r}$ indicates that the region with adequate signal quality is enlarged, which is the coverage improvement referred to in this paper.

## 4. Simulation Analysis and Measurement Validation

Using roadside trees as a representative case, this section quantitatively analyzes the influence of vegetation on key channel characteristics such as multipath structure and received power through a combination of simulation and measurement [24]. First, the modulation effect of vegetation on signal propagation paths is quantitatively revealed from the multipath delay and angular domains. Then, the influence patterns of frequency and canopy size on received power are explored. Finally, the effectiveness of the proposed method is validated through measured data from a campus tree-lined avenue scenario.

To make the simulation and measurement results reproducible, the common protocol adopted in this section is summarised here. The prediction plane is sampled on a uniform 1.0 m by 1.0 m grid at a height of 2.0 m above the ground. The angular discretisation of the transmitting ray tubes is chosen so that the spatial resolution of the resulting ray density matches the receiver grid spacing at the farthest prediction distance. The angular discretisation of the vegetation BSDF is 6° for the incident direction and 1° for the scattering direction. Reflection, diffraction, and diffuse scattering are handled by the standard Fresnel coefficient, the uniform geometrical theory of diffraction, and the directive scattering model, respectively, as detailed in [18]. The boundary condition at the outer edge of the prediction plane is the free-space continuation of the ray tube, and the prediction plane is extended well beyond the region of interest to avoid edge artefacts. The total radiated power, the carrier frequency, the antenna heights, and the prediction grid spacing used in each simulation case are listed in the corresponding subsection below. For the measurement campaign, the measurement setup and procedure follow those of our previous RT-MDS study [18].

For completeness, the physical parameters adopted in the multipath analysis of Section 4.1, covering both the LoS and NLoS scenarios, are summarised in Table 1.

**Figure 8:**
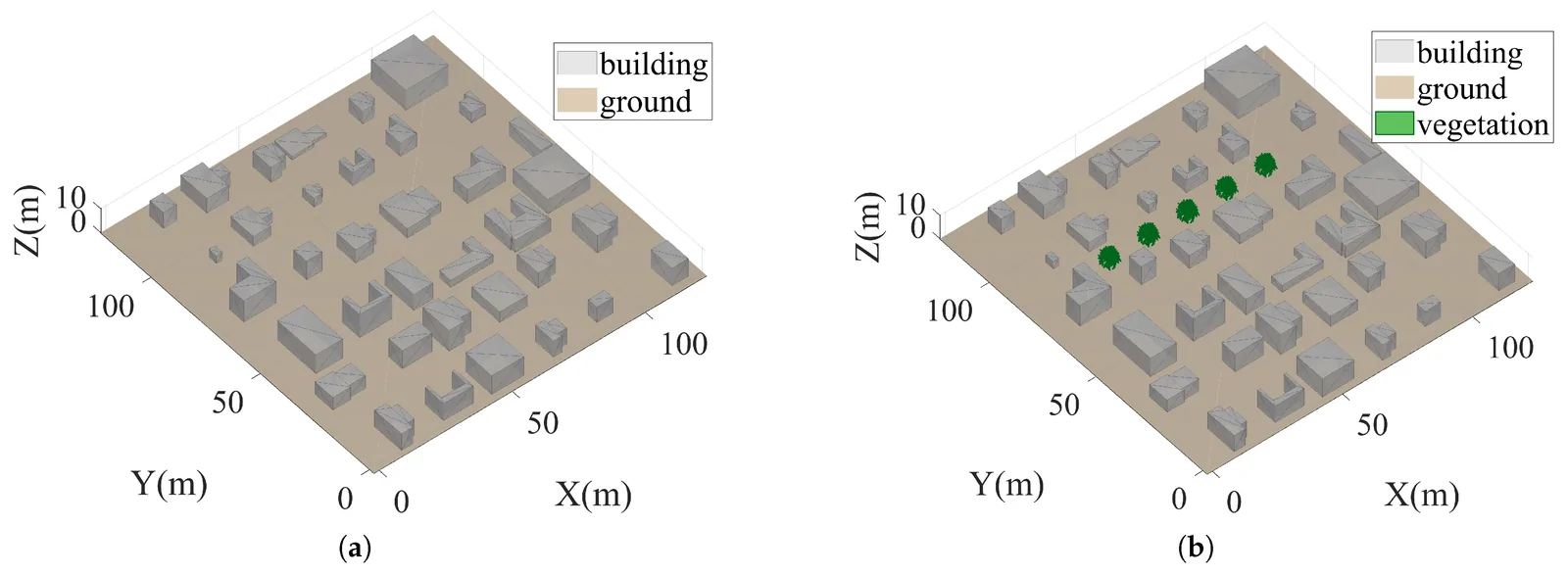
Schematic diagram of simulation scenarios for evaluating vegetation influence on channels: (**a**) without vegetation; (**b**) with vegetation.

**Figure 9:**
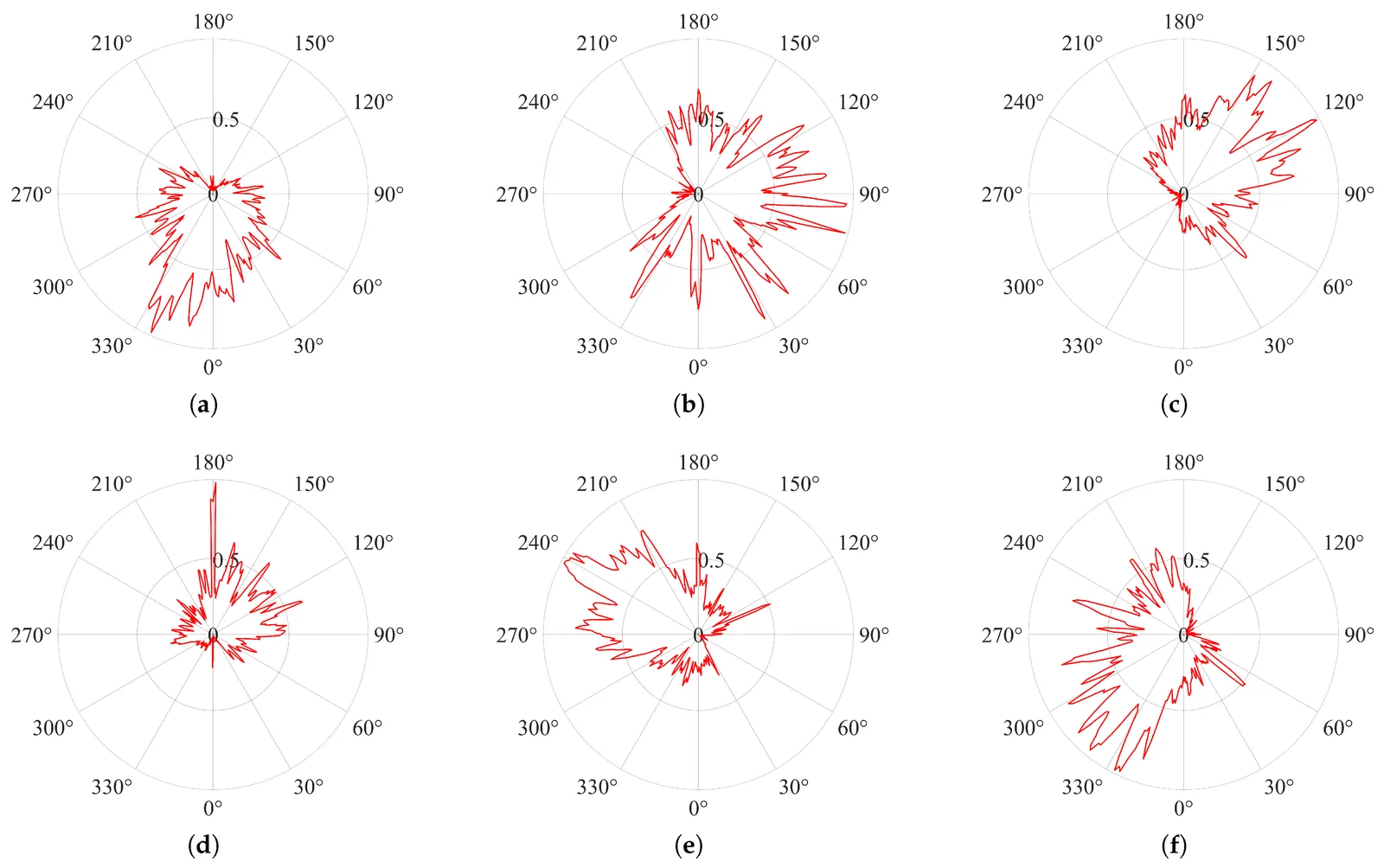
Vegetation BSDF scattering patterns under different incident angles: (**a**) 0°; (**b**) 60°; (**c**) 120°; (**d**) 180°; (**e**) 240°; (**f**) 300°.

The simulation scenarios are shown in Figure 8, including buildings, ground surface, and vegetation. The building and ground triangular facets number approximately 800, while the five trees encompass approximately 150,000 triangular facets. Consequently, using the fine structure of vegetation directly would significantly degrade the computational performance of the ray tracing program. In this simulation, the BSDF of each vegetation element is first computed and stored. For all subsequent simulations in this paper, the angular discretization interval of the incident direction is 6 degrees and that of the scattering direction is 1 degree. Subsequently, four pieces of information, including the BSDF, geometric center, radius, and transmission electric field loss coefficient of each vegetation element, are passed to the simulation. The scattering coefficients of the trees used in the simulation under different incident directions in the horizontal plane are shown in Figure 9.

### 4.1. Modulation Effect of Vegetation on Multipath Structure

**Figure 10:**
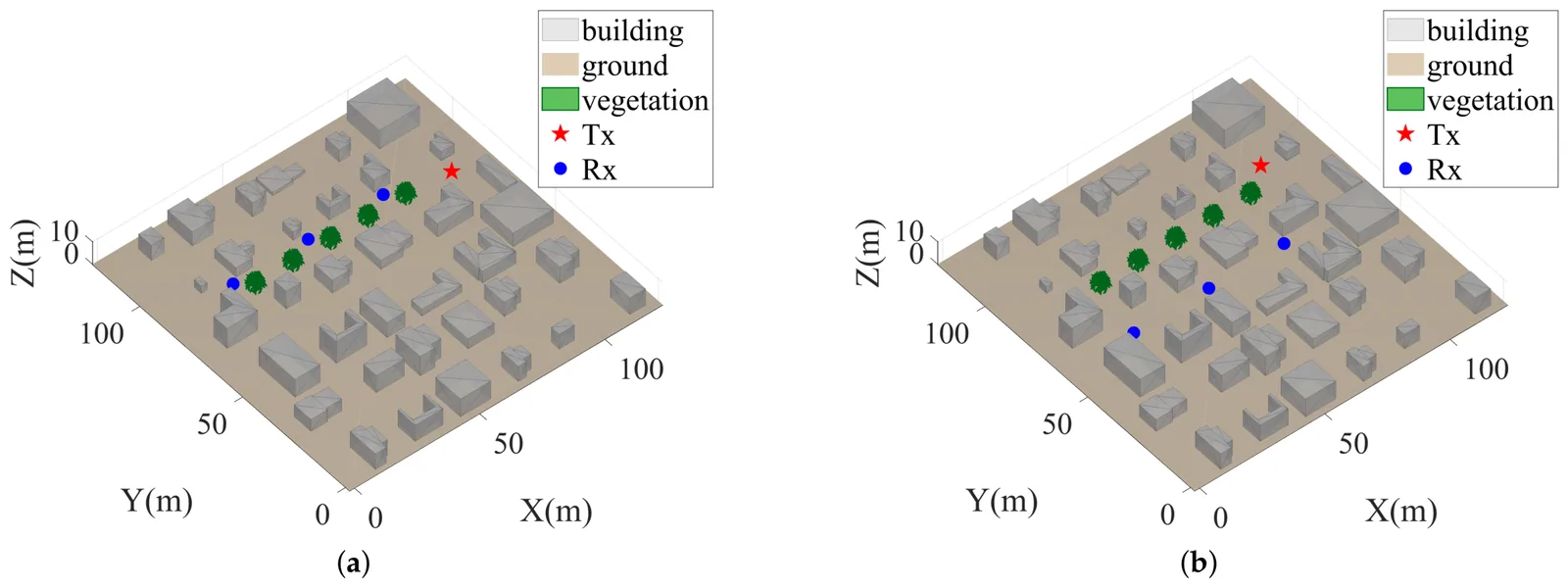
Schematic diagram of antenna positions in the simulation: (**a**) LoS; (**b**) NLoS.

To investigate the influence of vegetation on multipath, the simulation is configured with the physical parameters summarised in Table 1, and the antenna placement for the line-of-sight (LoS) and non-line-of-sight (NLoS) conditions is shown in Figure 10.

**Figure 11:**
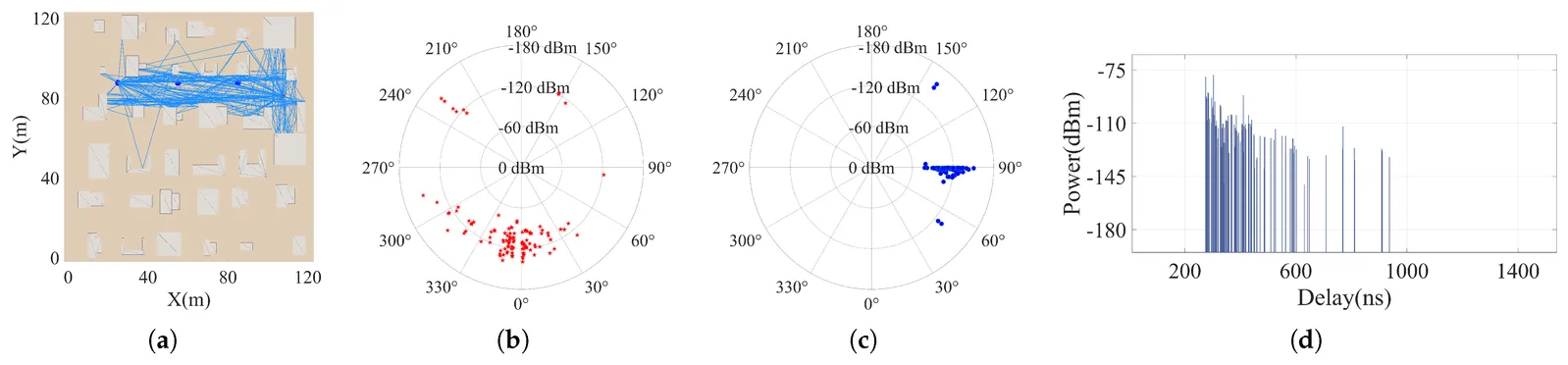
Multipath simulation results for Rx11 without vegetation: (**a**) spatial multipath (121 paths); (**b**) APS; (**c**) EPS; (**d**) PDP.

**Figure 12:**
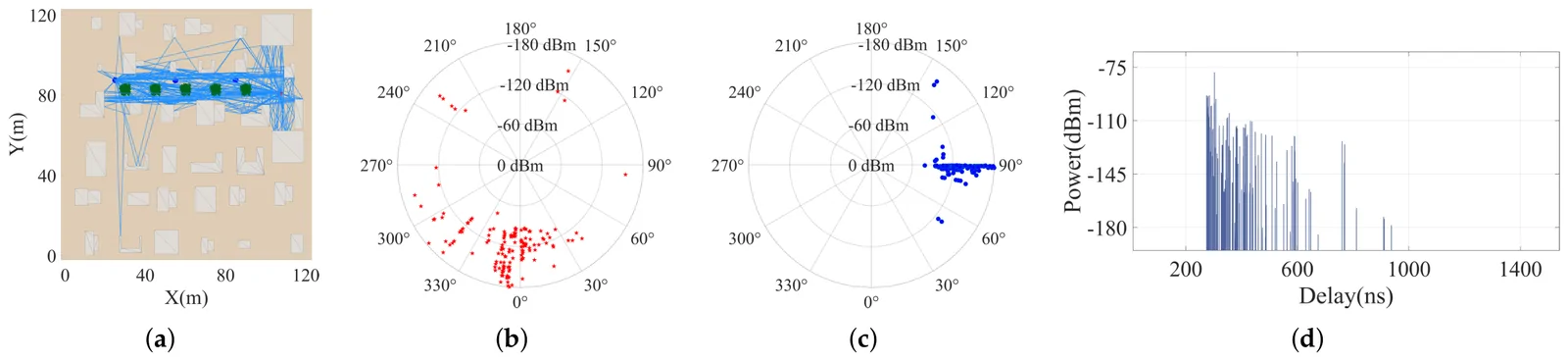
Multipath simulation results for Rx11 with vegetation: (**a**) spatial multipath (165 paths); (**b**) APS; (**c**) EPS; (**d**) PDP.

**Figure 13:**
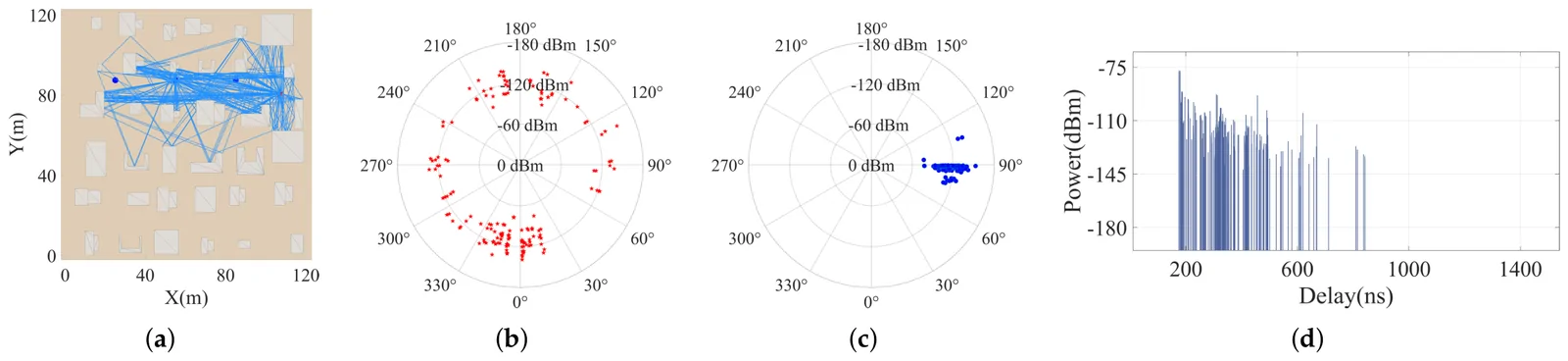
Multipath simulation results for Rx12 without vegetation: (**a**) spatial multipath (157 paths); (**b**) APS; (**c**) EPS; (**d**) PDP.

**Figure 14:**
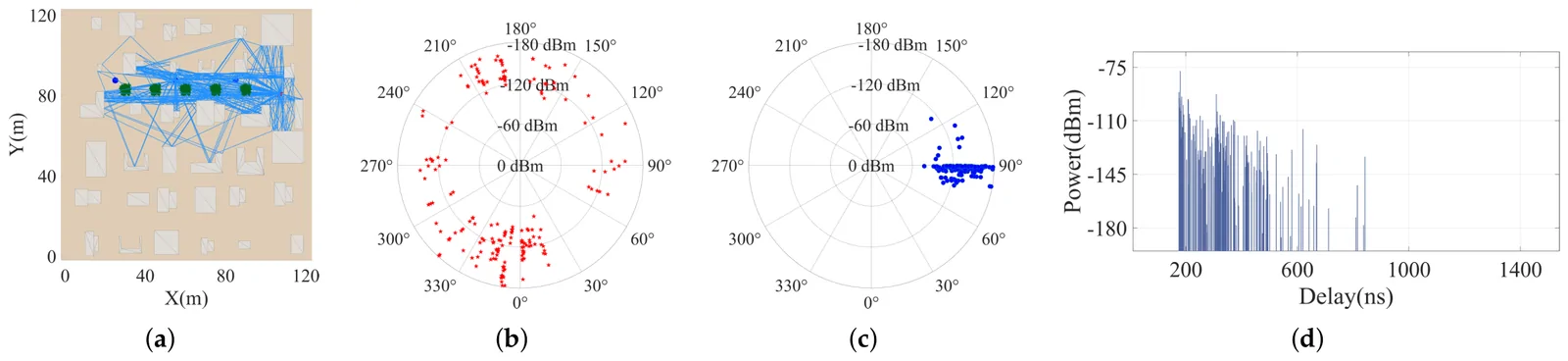
Multipath simulation results for Rx12 with vegetation: (**a**) spatial multipath (198 paths); (**b**) APS; (**c**) EPS; (**d**) PDP.

**Figure 15:**
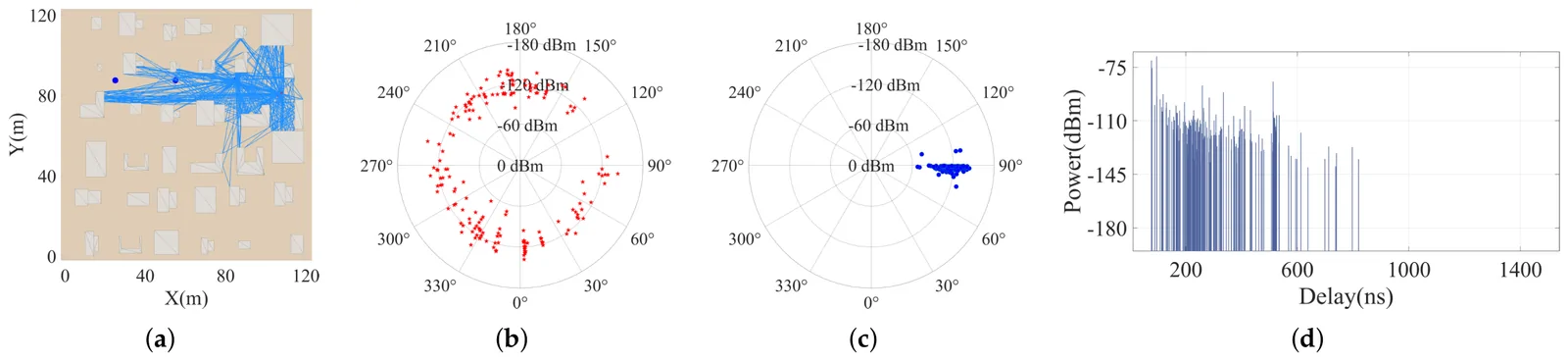
Multipath simulation results for Rx13 without vegetation: (**a**) spatial multipath (187 paths); (**b**) APS; (**c**) EPS; (**d**) PDP.

**Figure 16:**
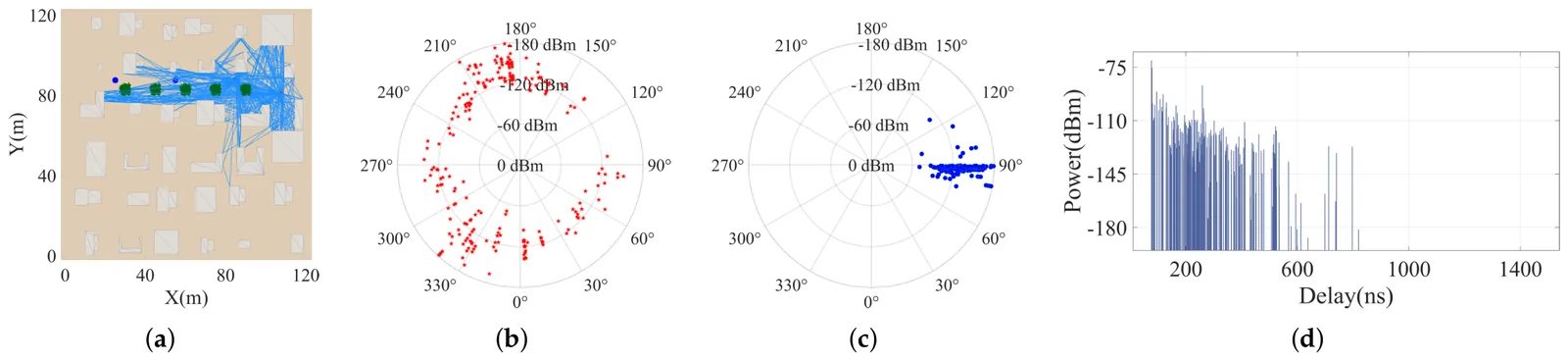
Multipath simulation results for Rx13 with vegetation: (**a**) spatial multipath (232 paths); (**b**) APS; (**c**) EPS; (**d**) PDP.

**Figure 17:**
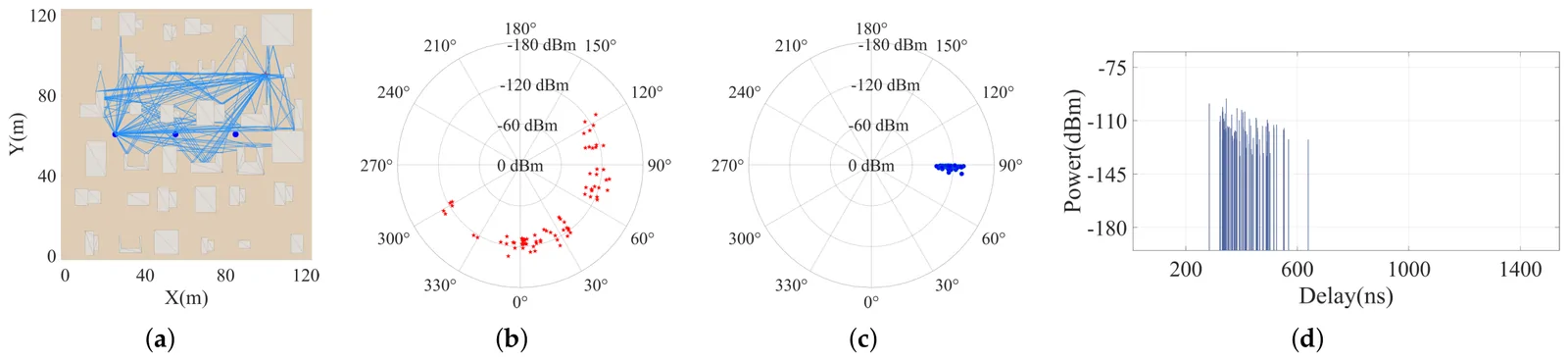
Multipath simulation results for Rx21 without vegetation: (**a**) spatial multipath (80 paths); (**b**) APS; (**c**) EPS; (**d**) PDP.

**Figure 18:**
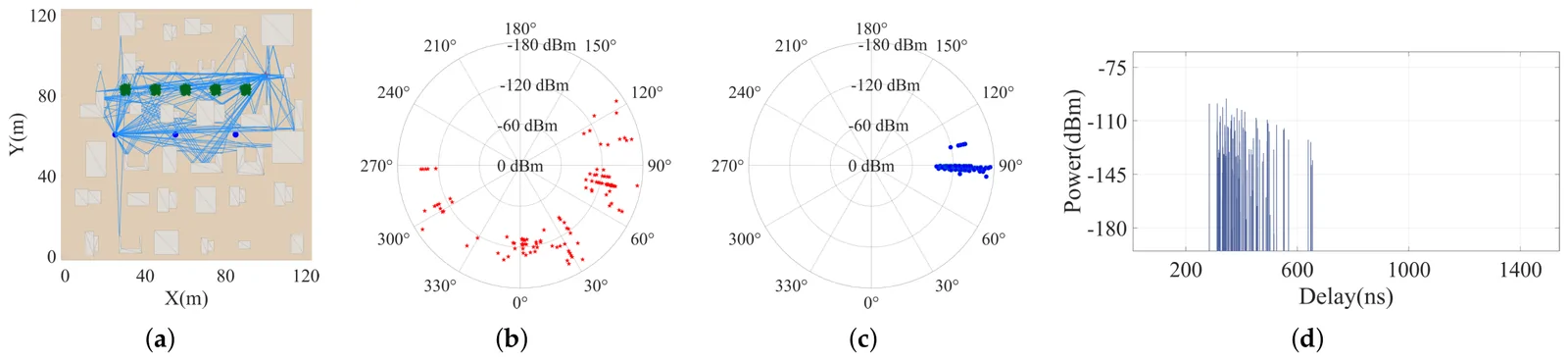
Multipath simulation results for Rx21 with vegetation: (**a**) spatial multipath (122 paths); (**b**) APS; (**c**) EPS; (**d**) PDP.

**Figure 19:**
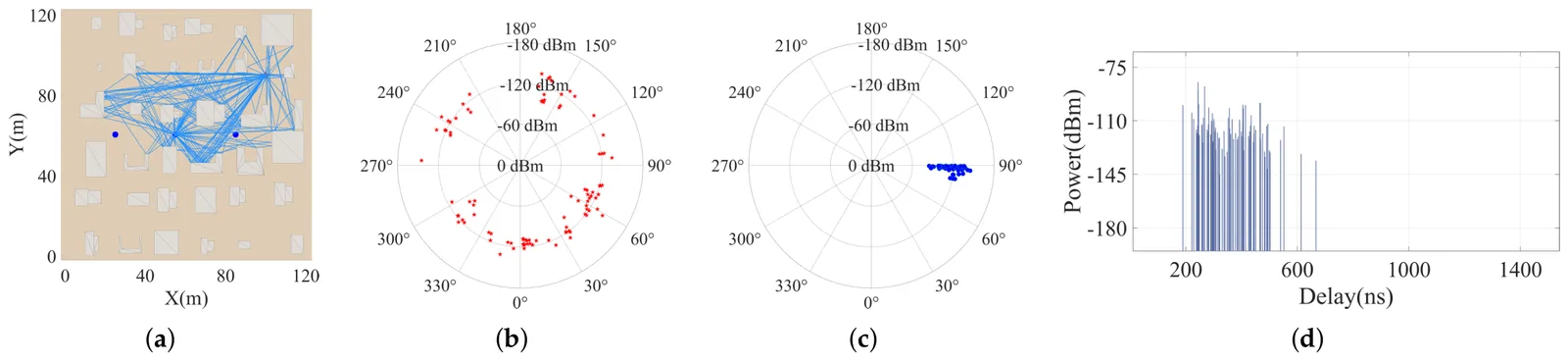
Multipath simulation results for Rx22 without vegetation: (**a**) spatial multipath (101 paths); (**b**) APS; (**c**) EPS; (**d**) PDP.

**Figure 20:**
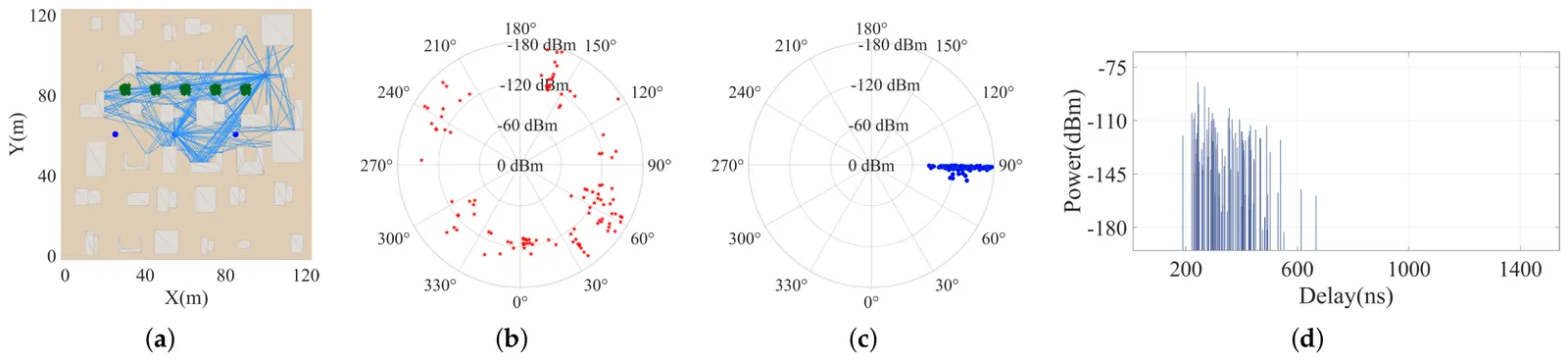
Multipath simulation results for Rx22 with vegetation: (**a**) spatial multipath (121 paths); (**b**) APS; (**c**) EPS; (**d**) PDP.

**Figure 21:**
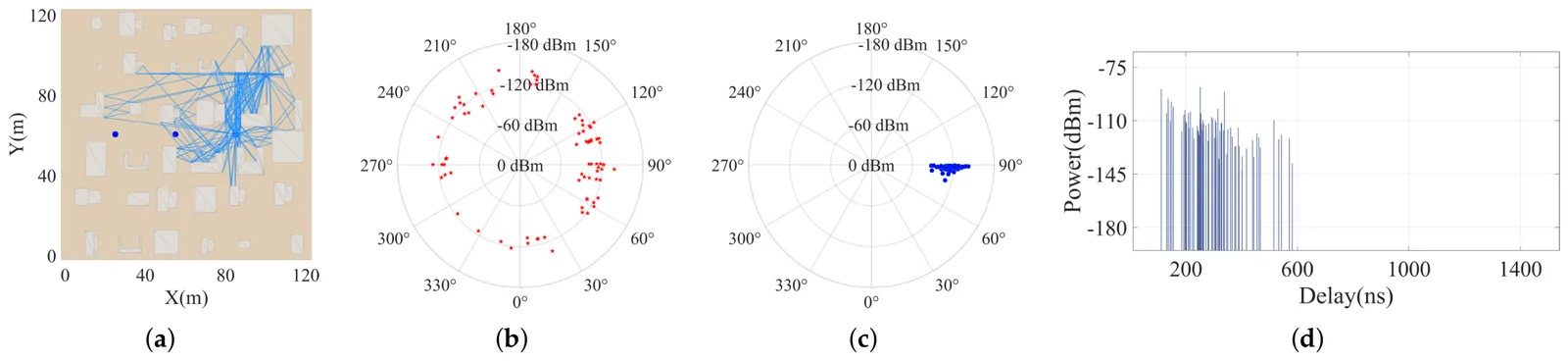
Multipath simulation results for Rx23 without vegetation: (**a**) spatial multipath (80 paths); (**b**) APS; (**c**) EPS; (**d**) PDP.

**Figure 22:**
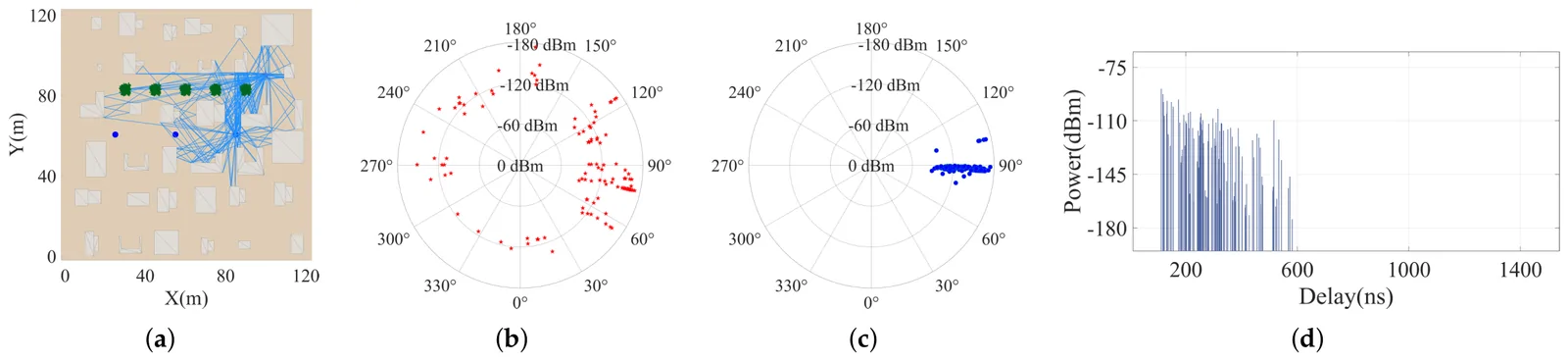
Multipath simulation results for Rx23 with vegetation: (**a**) spatial multipath (117 paths); (**b**) APS; (**c**) EPS; (**d**) PDP.

Figure 11, Figure 12, Figure 13, Figure 14, Figure 15, Figure 16, Figure 17, Figure 18, Figure 19, Figure 20, Figure 21 and Figure 22 present the multipath simulation results for the LoS positions (Rx11, Rx12, Rx13) and NLoS positions (Rx21, Rx22, Rx23), each with and without vegetation. Each figure comprises four panels: (**a**) the spatial multipath distribution, (**b**) the azimuth power spectrum of arrival (APS), (**c**) the elevation power spectrum of arrival (EPS), and (**d**) the power delay profile (PDP).

From the perspective of physical mechanisms, vegetation affects multipath propagation through two pathways: first, penetration attenuation, which occurs when rays traverse the tree canopy, where the complex permittivity of the vegetation medium causes ohmic losses, with power decreasing exponentially with traversal distance; second, BSDF scattering, which occurs when rays collide with vegetation center points, generating numerous low-power scattering paths in all angular directions as described by the BSDF. These two mechanisms act in opposite directions, and the net effect of vegetation is determined by their competition and balance.

As shown in Figure 11, Figure 12, Figure 13, Figure 14, Figure 15 and Figure 16, under LoS conditions, the main path is suppressed and the tail is elevated. If the direct path traverses the canopy, it suffers penetration attenuation, shifting the main peak of the power delay profile downward. BSDF scattering paths form a continuous scattering tail after the main peak, causing the power spectrum to exhibit bidirectional variation characteristics of a lowered main peak and an elevated tail. Multipath capacity expansion occurs alongside path annihilation. Numerous new scattering paths are introduced by BSDF, but penetration attenuation causes some low-power reflection or diffraction paths to fall below the reception threshold due to additional losses and become annihilated, with the net increase in multipath count being the difference between the two. The elevation and azimuth angle distributions evolve from discrete structures to continuous uniform spectra driven by BSDF, improving angular diversity. Range dependency is significant; as the Tx-Rx distance increases, the probability and length of the direct path traversing the canopy both increase, making the cumulative effect of penetration loss more pronounced. BSDF scattering paths have already undergone substantial power attenuation due to long propagation distances, and their positive contribution is relatively weakened. Therefore, in the far-field region, the net effect of vegetation tends toward negative attenuation.

As shown in Figure 17, Figure 18, Figure 19, Figure 20, Figure 21 and Figure 22, under NLoS conditions, the critical hole-filling effect is significant. With no direct path present, BSDF scattering paths become the primary source for filling signal gaps in shadow regions, transforming the net effect of vegetation from auxiliary enhancement in LoS scenarios to critical hole-filling. The annihilation effect of penetration attenuation is weakened. After multiple interactions, the power of reflection or diffraction paths is already substantially attenuated, and the additional loss from penetration attenuation constitutes a small fraction relative to the existing high path loss. Therefore, the addition of BSDF scattering paths almost purely translates into net growth in multipath count. Scattering paths become more significant. The power gap between BSDF scattering paths and penetration-attenuated reflection or diffraction paths narrows, with vegetation scattering path power levels approaching those of reflection paths, and their relative weights in maximum-ratio combining increase significantly. The power spectrum exhibits peak-shaving and valley-filling spectral modulation: penetration attenuation depresses residual reflection or diffraction pulse peaks, while BSDF scattering paths fill delay gap regions, resulting in a continuous envelope at an overall low power level that reduces the risk of frequency-selective fading. The angular distribution evolves from highly directional to nearly isotropic, significantly reducing the probability of communication outage caused by angle-selective fading.

### 4.2. Influence of Frequency on Received Power with Vegetation

The prediction plane height is 2.0 m, the prediction plane grid spacing is 1.0 m, and the simulation frequencies are 5 GHz, 10 GHz, and 15 GHz. Tx1, Tx2, and Tx3 are located at (100 m, 90 m, 3 m), (60 m, 60 m, 3 m), and (25 m, 35 m, 3 m), respectively.

**Figure 23:**
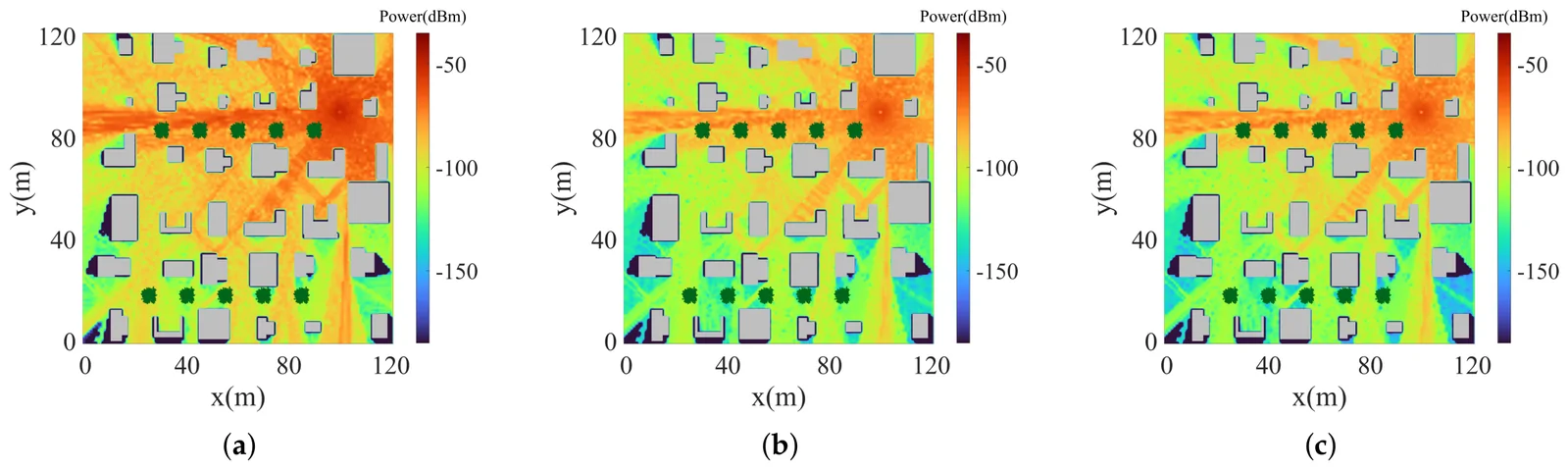
Coverage predictions for Tx1 at: (**a**) 5 GHz; (**b**) 10 GHz; (**c**) 15 GHz.

**Figure 24:**
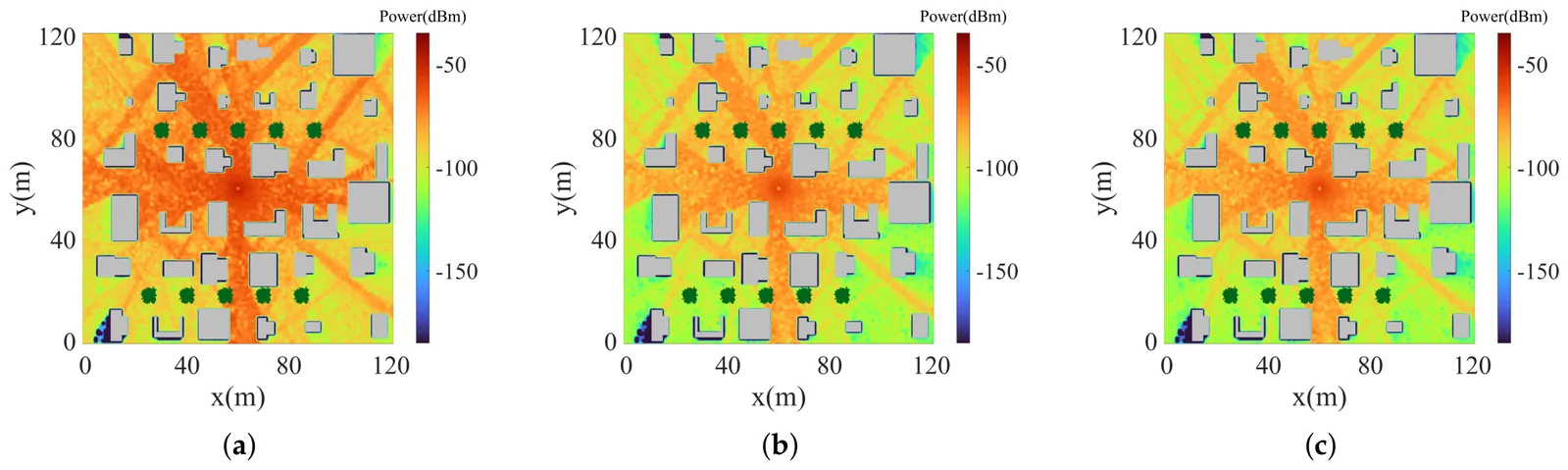
Coverage predictions for Tx2 at: (**a**) 5 GHz; (**b**) 10 GHz; (**c**) 15 GHz.

**Figure 25:**
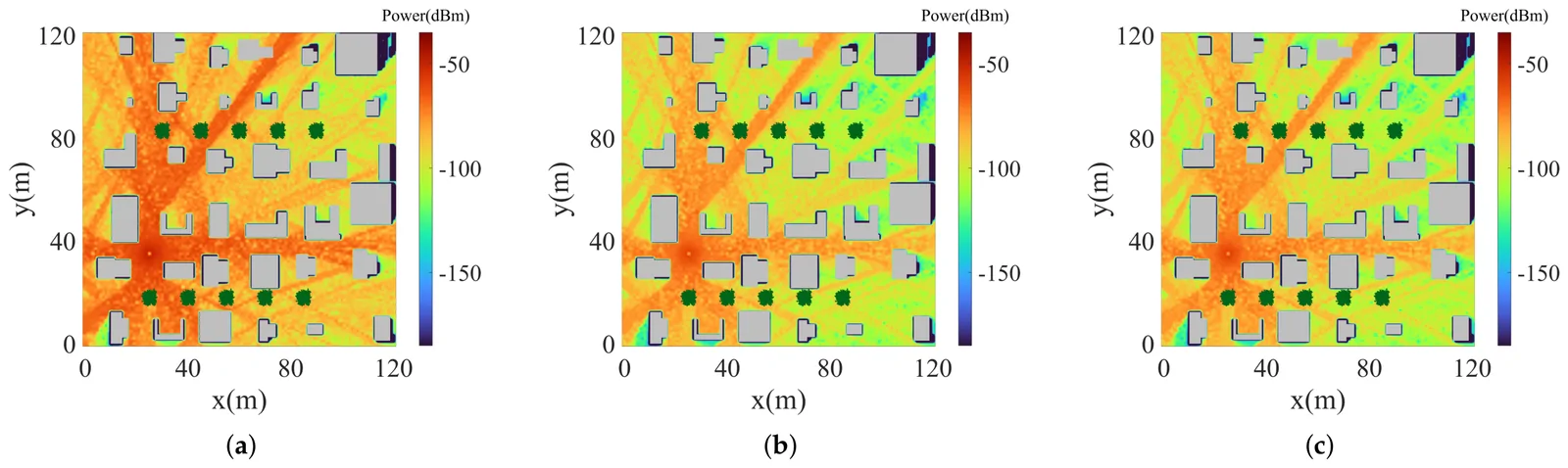
Coverage predictions for Tx3 at: (**a**) 5 GHz; (**b**) 10 GHz; (**c**) 15 GHz.

Other simulation parameters are identical to those described previously. The simulation results for Tx1, Tx2, and Tx3 are shown in Figure 23, Figure 24, and Figure 25, respectively, and the relevant statistical results are summarized in Table 2.

From Figure 23, Figure 24 and Figure 25, and Table 2, the following can be observed.

As frequency increases, the mean received power exhibits a clear downward trend. For instance, for Tx1, the power decreases by approximately 9.61 dB from 5 GHz to 10 GHz, but only by approximately 0.71 dB from 10 GHz to 15 GHz, showing significant nonlinearity. This phenomenon arises from two superimposed factors: free-space path loss is proportional to the square of frequency, with the frequency doubling from 5 to 10 GHz contributing approximately 6 dB of loss; and vegetation penetration and BSDF scattering losses slow their growth rate at higher frequencies due to dielectric dispersion, causing the total loss to approach saturation.

The coverage ratio (received power > −160 dBm) does not vary significantly with frequency, with fluctuations not exceeding 0.3 percentage points at any location. The fundamental reason lies in the fact that BSDF scattering is essentially an energy redistribution mechanism, in which incident energy is reradiated in all spatial directions. The breadth of coverage is dominated by vegetation geometry and dielectric properties with small frequency dependence, whereas the strength of coverage (mean power) decreases as frequency increases. Different Tx positions exhibit different sensitivity to frequency; the total decrease from 5 GHz to 15 GHz is smallest for Tx3 (9.21 dB, 1.10 dB less than Tx1), which may be related to the lighter overlap between the LoS path of Tx3 and the vegetation canopy, with BSDF scattering paths contributing a higher proportion and thus maintaining better coverage performance.

### 4.3. Influence of Vegetation Canopy Size on Received Power

**Figure 26:**
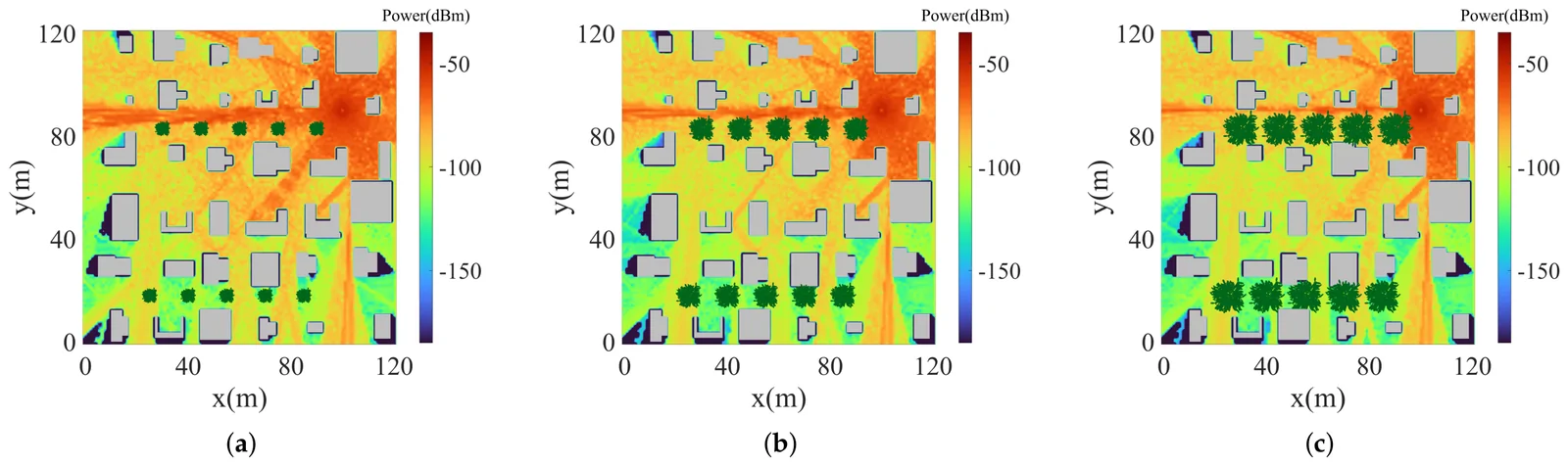
Coverage predictions for Tx1 at: (**a**) 3 m; (**b**) 5 m; (**c**) 7 m.

**Figure 27:**
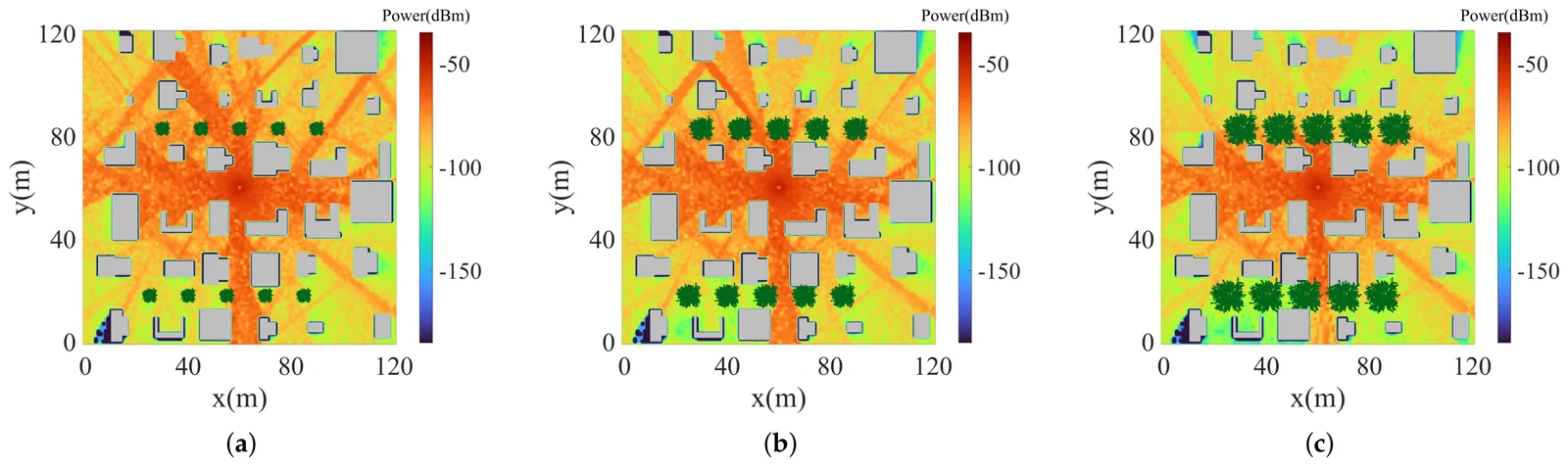
Coverage predictions for Tx2 at: (**a**) 3 m; (**b**) 5 m; (**c**) 7 m.

**Figure 28:**
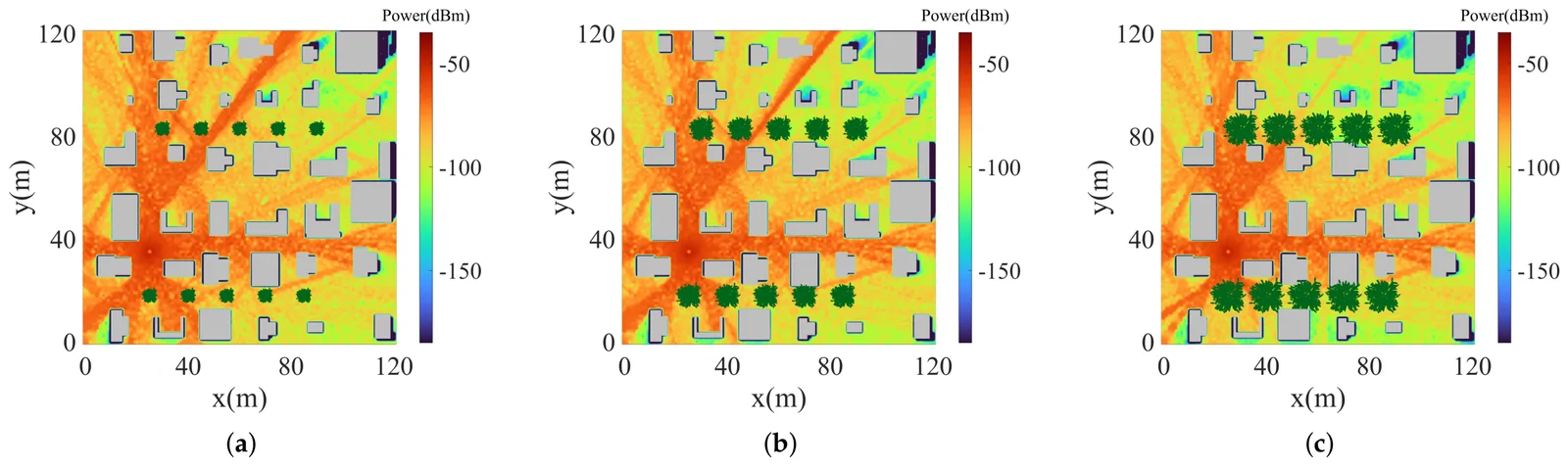
Coverage predictions for Tx3 at: (**a**) 3 m; (**b**) 5 m; (**c**) 7 m.

Vegetation canopy size is an important geometric parameter for characterizing vegetation. In this paper, it is approximated as a sphere, with radius being the primary dimension parameter. To investigate the influence of this parameter on received power, the simulation frequency is set to 5 GHz, and individual vegetation radii are set to 3 m, 5 m, and 7 m, with all other simulation parameters identical to those described previously. The simulation results for Tx1, Tx2, and Tx3 are shown in Figure 26, Figure 27, and Figure 28, respectively, and the relevant statistical results are summarized in Table 3.

From Figure 26, Figure 27 and Figure 28, and Table 3, the following can be observed.

As the vegetation radius increases, the mean received power exhibits a clear downward trend. For instance, for Tx1, the power decreases by approximately 2.46 dB from 3 m to 5 m and by approximately 1.07 dB from 5 m to 7 m. The physical mechanism of power reduction encompasses two aspects: the increasing canopy radius elevates both the probability and path length of rays traversing the vegetation medium, with penetration loss growing exponentially with traversal distance; simultaneously, the increased radius alters the energy distribution of the BSDF scattering pattern, directing more energy toward directions deviating from the Rx.

The coverage ratio decreases slightly with increasing radius, but the decline is far smaller than that of mean power (only 0.03 to 0.30 percentage points at each location). This phenomenon reflects the coverage maintenance characteristic of BSDF scattering: while increasing radius augments penetration loss, it also provides a larger scattering cross-section to reradiate energy into broader spatial angles, allowing coverage breadth to be relatively preserved.

Different Tx positions exhibit varying sensitivity to radius. Tx2 shows the largest total decrease (4.92 dB) from 3 m to 7 m, yet its absolute power level consistently remains the highest (−130.99 to −135.91 dBm). This may be related to the fact that the LoS path of Tx2 does not traverse the core region of the canopy even as the radius increases, with penetration loss primarily affecting reflection paths rather than the direct main path, making the power decrease relatively moderate.

### 4.4. Large-Scale Channel Measurement and Simulation Analysis

**Figure 29:**
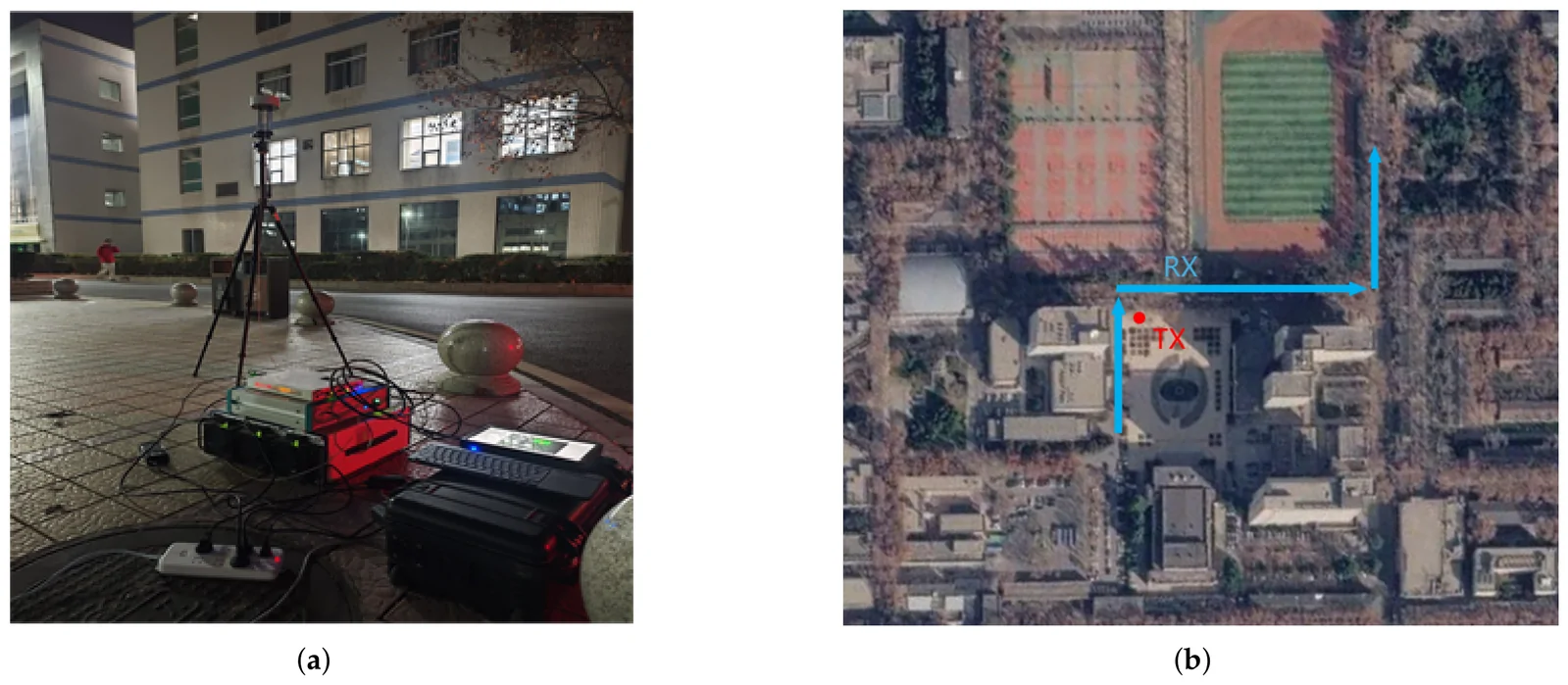
Schematic diagram of antenna positions in the measurement scenario: (**a**) LoS; (**b**) NLoS.

To validate the large-scale channel prediction accuracy of the HFB-RTT-3D model in typical outdoor scenarios containing vegetation, and to evaluate the improvement in prediction performance brought by vegetation BSDF scattering modeling, this paper selects a tree-lined avenue on a university campus as the test scenario. As shown in Figure 29, this scenario encompasses both LoS and NLoS propagation conditions: when the Rx is located in canopy gap regions, direct paths exist; when the Rx is directly beneath the canopy or obstructed by it, the direct path is blocked by vegetation, and signals can only reach the Rx through non-direct paths such as reflection, diffraction, and vegetation scattering. This typical urban environment with mixed vegetation and building propagation represents an ideal scenario for testing the ray tracing model’s capability in modeling vegetation scattering effects.

Fine geometric modeling of the tree-lined avenue scenario is performed using three-dimensional modeling software. During the modeling process, conventional environmental elements such as building exteriors, road surfaces, and lawns are constructed according to actual dimensions, while roadside trees are generated using the procedural modeling method described in Section 2.2.

**Figure 30:**
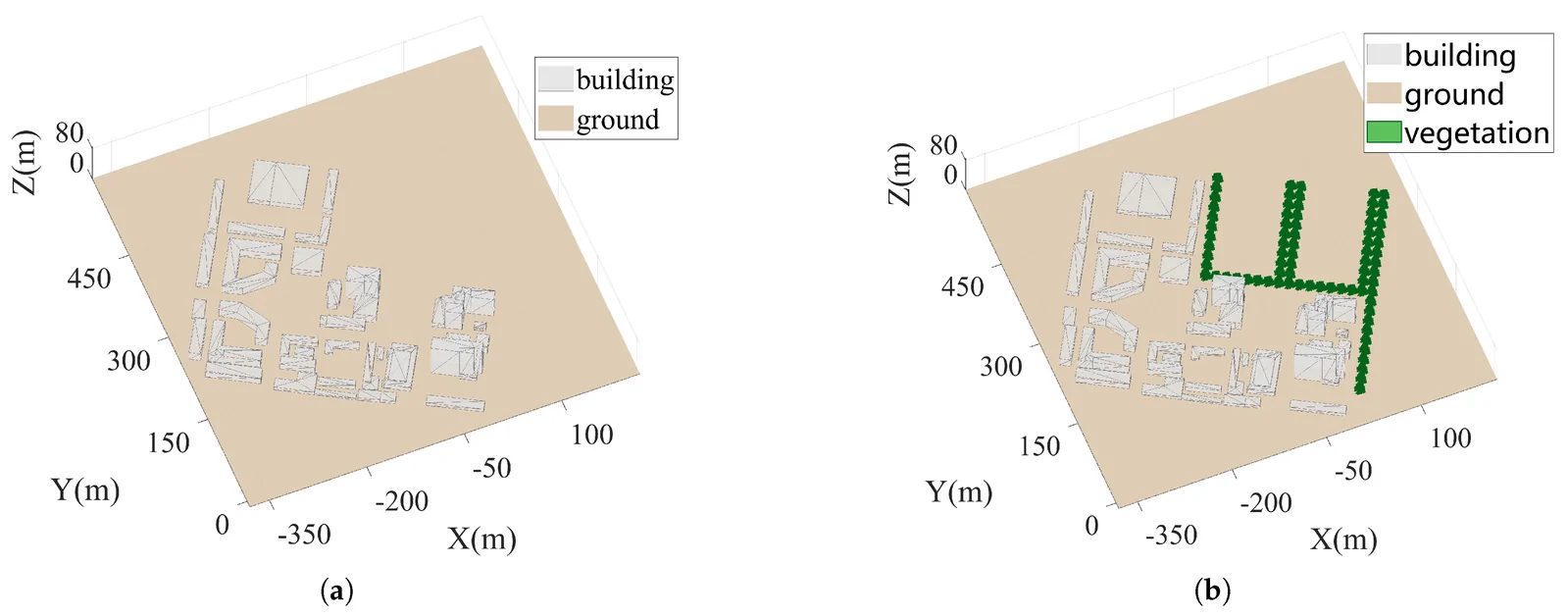
Three-dimensional environmental model: (**a**) LoS perspective; (**b**) NLoS perspective.

The electromagnetic parameters of the trees are set according to ITU-R Recommendation P.833 [12] and the vegetation electromagnetic model in Section 2.2. The three-dimensional environmental model is shown in Figure 30. To evaluate the contribution of vegetation modeling to channel prediction accuracy, two comparison models are constructed in this paper: one that does not consider the influence of trees and one that does, with all other simulation parameters being completely identical.

**Figure 31:**
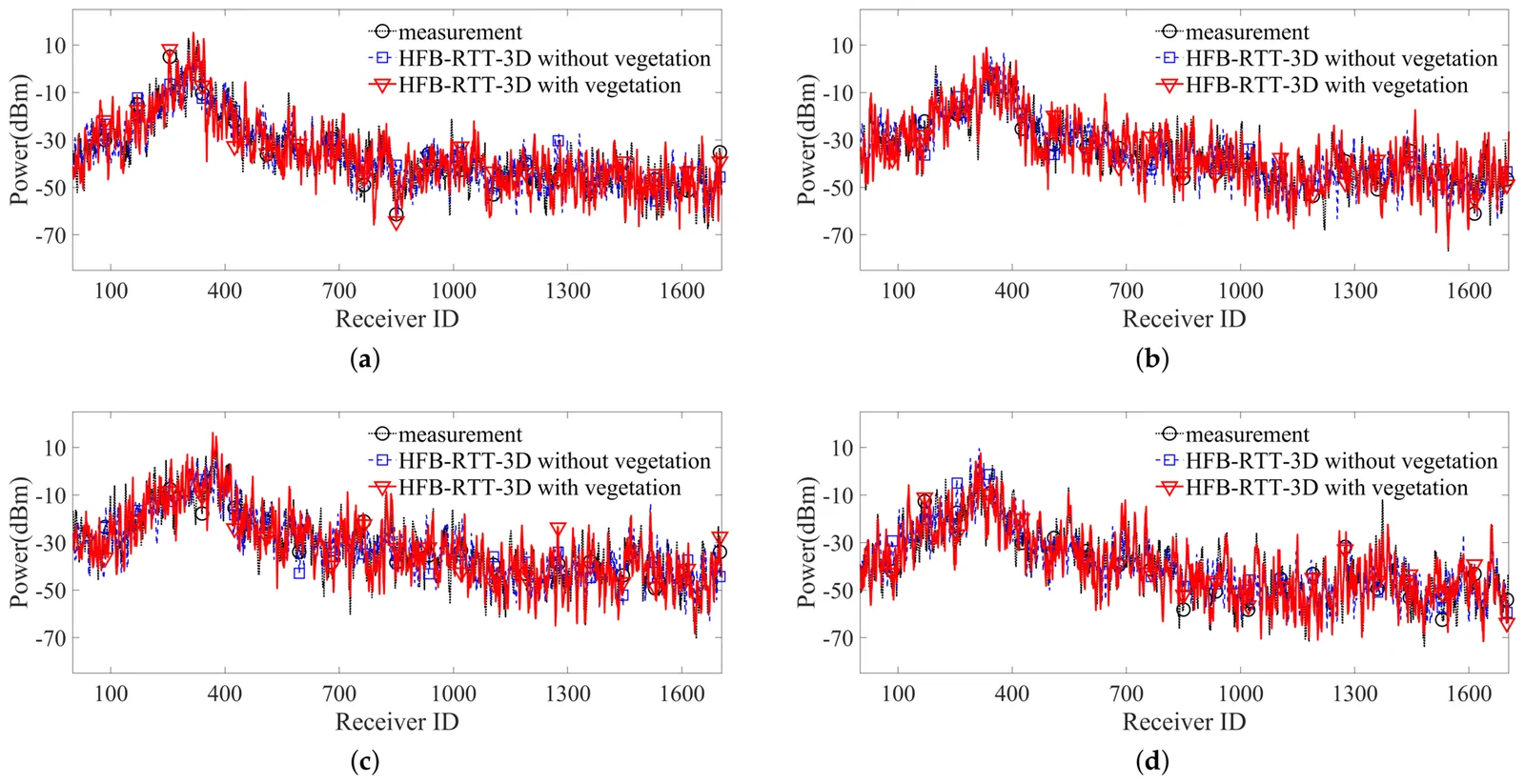
Comparison between simulation and measurement results: (**a**) 2.3 GHz; (**b**) 3 GHz; (**c**) 4 GHz; (**d**) 5.9 GHz.

The measurement process in this experiment is similar to the measurement activities in RT-MDS. A biconical omnidirectional antenna (model SZ-2001800/P) is employed as both the transmitting and the receiving antenna at all four frequency points, whose omnidirectional radiation in the horizontal plane is consistent with the isotropic antenna assumption adopted in the simulation. To systematically evaluate the vegetation scattering modeling effectiveness of the HFB-RTT-3D model across different frequency bands, four independent experiments are planned in this scenario, testing at four typical frequency points: 2.3 GHz, 3 GHz, 4 GHz, and 5.9 GHz. The comparison between the measured received power and simulation results for the four experiments is shown in Figure 31. To quantitatively evaluate the prediction accuracy of the model, root mean square error (RMSE) is adopted as the primary evaluation metric, and the RMSE values at the four frequency points are summarized in Table 4.

From Figure 31 and Table 4, it can be observed that the simulation results considering trees consistently demonstrate better agreement with measured data than those without trees, with accuracy improvements ranging from 0.57 to 1.67 dB. When trees are considered, the RMSE exhibits minimal fluctuation across the four frequency bands (maximum only 0.19 dB), indicating that the BSDF scattering mechanism possesses good frequency robustness. In the absence of vegetation, the RMSE increases and then decreases with frequency, reaching a peak of 7.97 dB at 4 GHz. This behavior can be attributed to the following: at the low-frequency band (2.3 GHz), vegetation scattering effects are weak, and the error introduced by neglecting trees is limited; as the frequency rises to 3–4 GHz, the BSDF scattering contribution increases, and the prediction deviation caused by neglecting trees is correspondingly amplified; at the 5.9 GHz band, free-space path loss increases by approximately 8.2 dB compared with 2.3 GHz, and the received power in vegetation shadow regions approaches the noise floor, with increased measurement uncertainty limiting the RMSE increment. In terms of propagation mechanisms, the improvement in prediction accuracy from tree modeling primarily originates from the signal supplementation provided by BSDF scattering in NLoS shadow regions, correcting the overestimation of predicted power that occurs when trees are neglected. In summary, the measurement validation demonstrates that vegetation BSDF scattering modeling can stably control the prediction RMSE below 7 dB in the tree-lined avenue scenario, confirming the effectiveness of the model in this scenario.

### 4.5. Computational Complexity and Comparison with Alternative Vegetation Models

The computational efficiency of the proposed HFB-RTT-3D engine is governed by the number of ray tubes processed in the forward tree, the per-collision cost of the material library lookup, and the per-collision cost of the BSDF evaluation. Let *M* denote the number of transmitting ray tubes, *N* the number of receivers, *K* the number of vegetation centres in the scenario, *T* the maximum vegetation scattering depth, $A_{i}$ the number of BSDF incident directions, and $A_{s}$ the number of BSDF scattering directions. The per-receiver wall-clock time is then dominated by the term $M\cdot N\cdot K\cdot T\cdot A_{i}\cdot A_{s}$ in the vegetation-enabled simulation, while the corresponding term for an explicit vegetation model would be $M\cdot N\cdot N_{v}$ where $N_{v}$ is the total number of explicit vegetation facets, of the order of $1.5\times 10^{5}$ in the present scenario. The single-centre BSDF lookup therefore replaces the inner per-facet loop by a single material library access, which is the main source of the speed-up achieved by the equivalent-scatterer abstraction. The absolute wall-clock numbers measured on the specific hardware platform used in this study are deliberately reported in a companion technical report rather than in the present paper, because they depend strongly on the compiler flags, the instruction-set extensions, and the memory hierarchy of the specific machine, and would distract the reader from the propagation-physics focus of the journal version.

A qualitative comparison of the proposed BSDF-based vegetation model with three widely used alternatives is summarised in Table 5. The empirical attenuation model recommended by ITU-R P.833 [12] provides a frequency-dependent specific attenuation but ignores the directional redistribution of the scattered energy, so it cannot reproduce the angular diversity of the multipath. The two-dimensional ray-tracing model of Leonor et al. [13] captures the directional redistribution in the plane of incidence but is restricted to two dimensions and to a single ornamental plant species. The hybrid physics-data-driven model of Zhang et al. [8] is the closest in scope to the present work, but it is trained on a specific measurement campaign and its generalisation to other vegetation species, ages, and pruning conditions is not reported. The present BSDF-based approach, by contrast, inherits the generality of the three-dimensional ray-tracing engine, keeps the leaf-level electromagnetic parameters explicit, and reduces the explicit-geometry cost through a single equivalent centre per tree. A quantitative RMSE comparison with at least one of the three alternatives in the same campus scenario will be included in the companion technical report, because the additional measurement and simulation workload is not within the scope of the present paper.

The above analysis supports the generalisability of the proposed method in three aspects. First, the ray-tube data structure is independent of the carrier frequency and of the specific scenario, so the same engine can be re-used at millimetre-wave, sub-terahertz, and higher bands. Second, the vegetation equivalent-scatterer abstraction does not depend on the geometry of a particular tree species, and the BSDF library can be extended with new species without modifying the tracing core. Third, the BSDF pre-simulation is decoupled from the runtime tracing, so that more detailed canopy geometries can be incorporated into the BSDF at any time without changing the per-collision cost of the engine.

## 5. Conclusions

This paper has presented a deterministic channel modeling method for urban multi-factor environments based on a hybrid forward-backward ray tube tracing approach. Building upon the RT-MDS framework [18], the proposed HFB-RTT-3D method systematically extends the modeling capability to urban hotspot areas by introducing two new types of pyramid-shaped ray tubes, namely, diffraction ray tubes and vegetation scattering ray tubes, achieving collaborative modeling of four propagation mechanisms within a single ray tube tree framework: specular reflection, diffuse scattering, edge diffraction, and vegetation BSDF scattering.

The core achievements of this paper are summarized as follows. A unified ray tube tree construction framework has been established, integrating multiple propagation mechanisms within a single data structure and tracing algorithm, enabling deterministic prediction of multipath channel characteristics in complex urban environments containing multiple propagation elements. Parametric vegetation geometry and electromagnetic models have been developed, leveraging L-system-based procedural generation and frequency-dependent dielectric characterization to support systematic simulation studies of vegetation effects across different tree species, growth stages, and operating frequencies. The leaf-level BSDF scattering model achieves computationally feasible representation of the angular, frequency, and polarization dependencies of vegetation scattering while maintaining physical plausibility. The mathematical definitions of the coverage ratio and the mean received power are given in Section 3.5 and are used consistently in the tables of Section 4, so that the synthetic studies and the measurement validation are directly comparable.

Simulation analyses show that vegetation introduces an additional attenuation of approximately 2 to 10 dB in the considered simulation settings, depending on the carrier frequency and the canopy size, and that the vegetation-only attenuation, obtained by removing the free-space path loss, grows with frequency but at a slower rate than $f^{2}$ because the dielectric dispersion of the leaves saturates at the upper end of the considered band. Under NLoS conditions, the BSDF scattering paths fill the angular and delay gaps left by the blocked direct path, so that the received power in the shadow region is consistently higher than in the bare-environment case. Comparative analyses across different canopy radii reveal the significant modulation effect of vegetation geometric parameters on channel characteristics. Measurement validation in a campus tree-lined avenue scenario confirms the prediction accuracy advantage of the model incorporating vegetation, with RMSE improved by 0.57 to 1.67 dB over bare-environment simulation at frequency points from 2.3 to 5.9 GHz.

From a sustainability perspective, the deterministic engine proposed in this paper contributes to greener network planning and operation in two ways. First, by replacing a substantial fraction of the drive-test campaigns with a site-specific ray-based prediction, the carbon footprint associated with field measurements is reduced, especially for dense urban scenarios where drive-test campaigns are logistically expensive and time consuming. Second, the explicit knowledge of the multipath structure, including the vegetation-induced attenuation, enables the radio access network to be planned with a tighter link-budget margin, which in turn allows the transmit power of the base station to be reduced without sacrificing coverage. Both effects align with the broader objective of reducing the energy consumption of future 5G and 6G networks, and they are quantified in the companion technical report referenced in Section 4.5.

### 5.1. Limitations

The work presented in this paper has several limitations that should be acknowledged. The vegetation equivalent-scatterer abstraction, although physically justified in Section 3.3, is an approximation, and the single-centre representation cannot capture the multipath-rich response of a deeply layered canopy. The quantitative sensitivity analysis with different numbers and locations of equivalent centres per tree is identified as a separate study and is not included in the present paper, because it requires dedicated measurement campaigns on vegetation clusters with different ages, species, and pruning conditions. The current measurements cover only a single campus tree-lined avenue scenario and four frequency points between 2.3 and 5.9 GHz, so the generalisation to other scenario types, vegetation species, and frequency bands is not directly demonstrated by the present data. The absolute wall-clock numbers of the deterministic engine depend strongly on the hardware platform, the compiler flags, and the instruction-set extensions, and are therefore reported in a companion technical report rather than in the journal version. The analytical model used for the diffraction coefficient is the uniform geometrical theory of diffraction, which is known to lose accuracy in the deep shadow region immediately behind a wedge; a more accurate coefficient would be required for site-specific applications in dense urban canyons. Finally, the BSDF generation in this paper assumes a single, time-invariant vegetation geometry per tree, so that the wind-induced time-varying response of the canopy is not captured.

### 5.2. Future Work

The future research directions suggested by the present work include the following items. Extending the method to higher frequency bands such as millimetre-wave and sub-terahertz, with the corresponding re-tuning of the BSDF library and the diffraction coefficient, is the most direct extension. Introducing wind-induced time-varying vegetation models, in which the BSDF is treated as a random process parameterised by the wind speed, will allow the channel dynamic characteristics such as the fading autocorrelation and the Doppler spectrum to be reproduced. Integrating the deterministic propagation engine with system-level simulation tools will support end-to-end performance evaluation for fifth-generation (5G) and sixth-generation (6G) systems, including the link-adaptation, scheduling, and beam-management loops. Exploring machine-learning-assisted ray tracing acceleration techniques, such as neural importance sampling and learned acceleration structures, may achieve near-real-time channel prediction in dynamic urban environments. The quantitative sensitivity analysis on the vegetation equivalent-scatterer abstraction, the additional measurement campaigns at millimetre-wave frequencies, and the wall-clock benchmarking of the deterministic engine on standard hardware platforms are all planned as separate technical contributions that will accompany the journal version of this paper.

## Figures and Tables

**Table 1:** Physical parameters adopted in the multipath analysis of Section 4.1, covering both the LoS and NLoS scenarios.

Parameter	Value	Description
Carrier frequency	5.0 GHz	Common to all receivers in the LoS and NLoS sets.
Tx1 position (LoS)	(107.0 m, 81.0 m, 3.0 m)	Height 3.0 m above ground.
Tx2 position (NLoS)	(100.0 m, 90.0 m, 3.0 m)	Height 3.0 m above ground.
LoS receiver positions	(25, 55, 85) m, y=87.5 m	z=2.0 m, indexed Rx11, Rx12, Rx13.
NLoS receiver positions	(25, 55, 85) m, y=60.5 m	z=2.0 m, indexed Rx21, Rx22, Rx23.
Tx radiated power	1 mW (0 dBm)	Total isotropically radiated power.
Tx and Rx antenna model	Isotropic, unit gain	Linear vertical polarisation.
Vegetation canopy radius	2 m	Radius of each equivalent scatterer.
Vegetation elements	5	One scatterer per tree, see Section 3.3.
BSDF incident step	6°	Angular resolution of the incident grid.
BSDF scattering step	1°	Angular resolution of the scattering grid.
Prediction plane height	2.0 m	Same as Rx height.
Prediction grid spacing	1.0 m	Uniform Δx=Δy=1.0 m.

**Table 2:** Influence of frequency on coverage prediction with vegetation.

Antenna Position	Frequency (GHz)	Coverage Ratio >−160 dBm	Mean Rx Power (dBm)
Tx1	5	79.06%	−138.6682
	10	78.91%	−148.2770
	15	78.86%	−148.9827
Tx2	5	80.66%	−129.8741
	10	80.51%	−139.2779
	15	80.45%	−139.3447
Tx3	5	79.41%	−132.9537
	10	79.38%	−142.1036
	15	79.37%	−142.1622

**Table 3:** Influence of vegetation canopy radius on coverage prediction.

Antenna Position	Radius (m)	Coverage Ratio >−160 dBm	Mean Rx Power (dBm)
Tx1	3	79.01%	−139.8580
	5	78.85%	−142.3217
	7	78.71%	−143.3900
Tx2	3	80.65%	−130.9929
	5	80.63%	−133.2751
	7	80.50%	−135.9086
Tx3	3	79.41%	−134.4651
	5	79.39%	−136.6800
	7	79.38%	−138.5507

**Table 4:** RMSE between HFB-RTT-3D and measurement results in the tree-lined avenue scenario.

Frequency (GHz)	Trees Considered	RMSE (dB)
2.3	Yes	6.28
	No	6.85
3	Yes	6.11
	No	7.34
4	Yes	6.30
	No	7.97
5.9	Yes	6.17
	No	7.78

**Table 5:** Qualitative comparison of the proposed BSDF-based vegetation model with three widely used alternatives. The columns list, from left to right, the four candidate models. The rows list the criteria on which the comparison is performed.

Criterion	ITU-R P.833 [12]	Leonor et al. [13]	Zhang et al. [8]	This Work
Directional redistribution	No	2D only	Yes (data-driven)	Yes (BSDF)
Frequency dependence	Yes	Limited	Yes	Yes
Geometric dimensionality	N/A	2D ray-tracing	Hybrid	3D ray-tracing
Cross-species portability	Yes	No (ornamental)	Limited (training)	Yes
Computational footprint	Low	Medium	Medium	Medium

## Data Availability

The data that support the findings of this study are available by contacting the corresponding authors.
